# Computational Study of Driving Forces in ATSP, PDIQ, and P53 Peptide Binding: C=O···C=O Tetrel Bonding Interactions at Work

**DOI:** 10.1021/acs.jcim.3c00024

**Published:** 2023-04-04

**Authors:** Lijun Lang, Antonio Frontera, Alberto Perez, Antonio Bauzá

**Affiliations:** † Chemistry Department, University of Florida, Gainesville, Florida 32611, United States; ‡ Department of Chemistry, Universitat de les Illes Balears, Crta. de Valldemossa km 7.5, 07122 Palma, Baleares, Spain

## Abstract

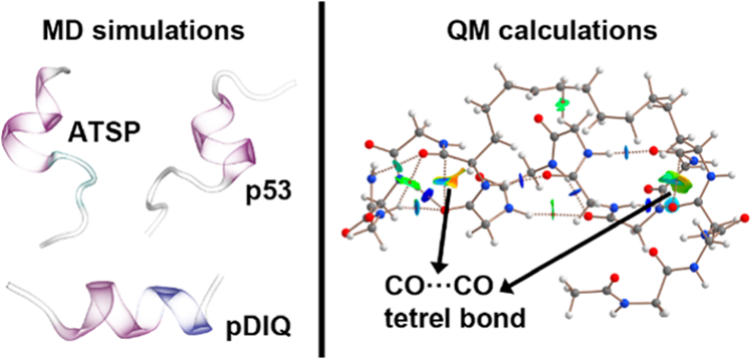

Understanding the molecular interactions that drive peptide folding is crucial to chemistry and biology. In this study, we analyzed the role of CO···CO tetrel bonding (TtB) interactions in the folding mechanism of three different peptides (ATSP, pDIQ, and p53), which exhibit a different propensity to fold in an α helix motif. To achieve this goal, we used both a recently developed Bayesian inference approach (MELDxMD) and Quantum Mechanics (QM) calculations at the RI-MP2/def2-TZVP level of theory. These techniques allowed us to study the folding process and to evaluate the strength of the CO···CO TtBs as well as the synergies between TtBs and hydrogen-bonding (HB) interactions. We believe that the results derived from our study will be helpful for those scientists working in computational biology, peptide chemistry, and structural biology.

## Introduction

Peptides are versatile molecules with applications in biology,^1^ drug discovery,^2^ nanomaterials,^3^ antimicrobials,^4^ and tissue engineering,^5^ to name a few. The wide range of functional groups of different sizes for each residue (20 considering only natural amino acids) and the large number of accessible backbone conformations make them suitable to bind at sites where other molecules (such as small, rigid molecules) cannot. Therefore, interest in pharmaceutical applications has steadily increased the development of peptide-based drugs, with several blockbusters such as Trulicity (diabetes) or Copaxone (multiple sclerosis) on the market.^6^ However, the large flexibility also raises some limitations: many peptides are intrinsically disordered in their free form^7^ and fold upon interacting with a complementary molecule (e.g., a protein binding site). Many of such interactions are transient, with low binding affinities and rapid off-rates, thus limiting their experimental characterization and computational prediction.^8^ In addition, their shorter length, compared to a protein, limits the amount of stabilizing intramolecular interactions. In this regard, some sequences are designed to increase the propensity for a particular conformation (typically folding into hairpins or helices). However, even these might have populations below 40% in solution. Many stabilizing forces, such as backbone hydrogen bonding to adopt secondary structure elements, can already be satisfied in solution with water molecules. The hydrophobic effect (desolvation) is often not enough to drive the peptide to stable conformations.^9^

In the presence of a binding partner, interactions through the backbone and side chains with the second molecule can lead to stable conformations (known as folding upon binding).^10^ In principle, we should be able to treat peptides as programmable molecules to accomplish self-assembly behavior or to identify peptides that bind with high affinity. However, computational tools based on force fields and other energy functions often lack the accuracy needed to study systems that lie at the edge of stability. Successful programming of peptides typically involves exploiting repeating patterns for self-assembly and, in cases of binding, maintaining the side chains responsible for interaction while increasing the propensity to adopt a secondary structure (e.g., through natural or non-natural amino acids such as chemical staples).^11,12^

The p53-MDM2 interaction is an important cancer target, where inhibiting the interaction would allow p53 to remain free in the cell and trigger apoptosis.^13^ The p53 epitope is intrinsically disordered and adopts a helical conformation upon binding MDM2 and the homologous MDMX14.^10^ MDM2 has a deep hydrophobic cavity, where three hydrophobic residues (Phe1 9,Trp23, and Leu26) from the peptide anchor themselves, adopting a helical conformation upon binding. Peptides pDIQ and ATSP have been proposed as potential inhibitors of the MDM2 protein since they retain the same interaction pattern while changing the sequence to increase helicity, therefore mimicking the p53-MDM2 binding mode. Concretely, peptide pDIQ^14^ adopts high populations of helical conformation states in its free form. At the same time, the ATSP peptide^11^ incorporates a chemical staple through a non-natural amino acid sequence to further increase helicity and uses an additional non-natural amino acid for anchoring during the binding event (Cba26).

In this study, we have combined classical and quantum mechanics calculations to analyze the folding patterns of p53, pDIQ, and ATSP peptides and the different backbone interactions that stabilize helical states beyond the well-known 1 → 4 hydrogen bonding (HB).^15^ Particularly, we have focused our attention on intramolecular interactions involving CO groups from the peptide backbone. This type of noncovalent force involves the interaction between a lone pair of electrons from an oxygen atom and the antibonding π orbital of a subsequent carbonyl group in the ground state, and previous studies have demonstrated its abundance and importance in protein folding and functionality.^16−18^ This interaction can also be described as a Tetrel bond (an attractive noncovalent force between a σ-/π-hole located in a group IV atom and a Lewis base).^19−21^ Tetrel bonds (TtBs), which typically involve a σ-hole located on an element from group IV and a Lewis base, were theoretically described by the groups of Frontera^19^ and Arunan in 2013.^20^ From these initial studies, several computational and experimental works have analyzed the physical nature of the interaction as well as its impact in the fields of supramolecular chemistry,^7,22^ crystal engineering,^23−25^ and biology.^26−28^

**Figure 1:**
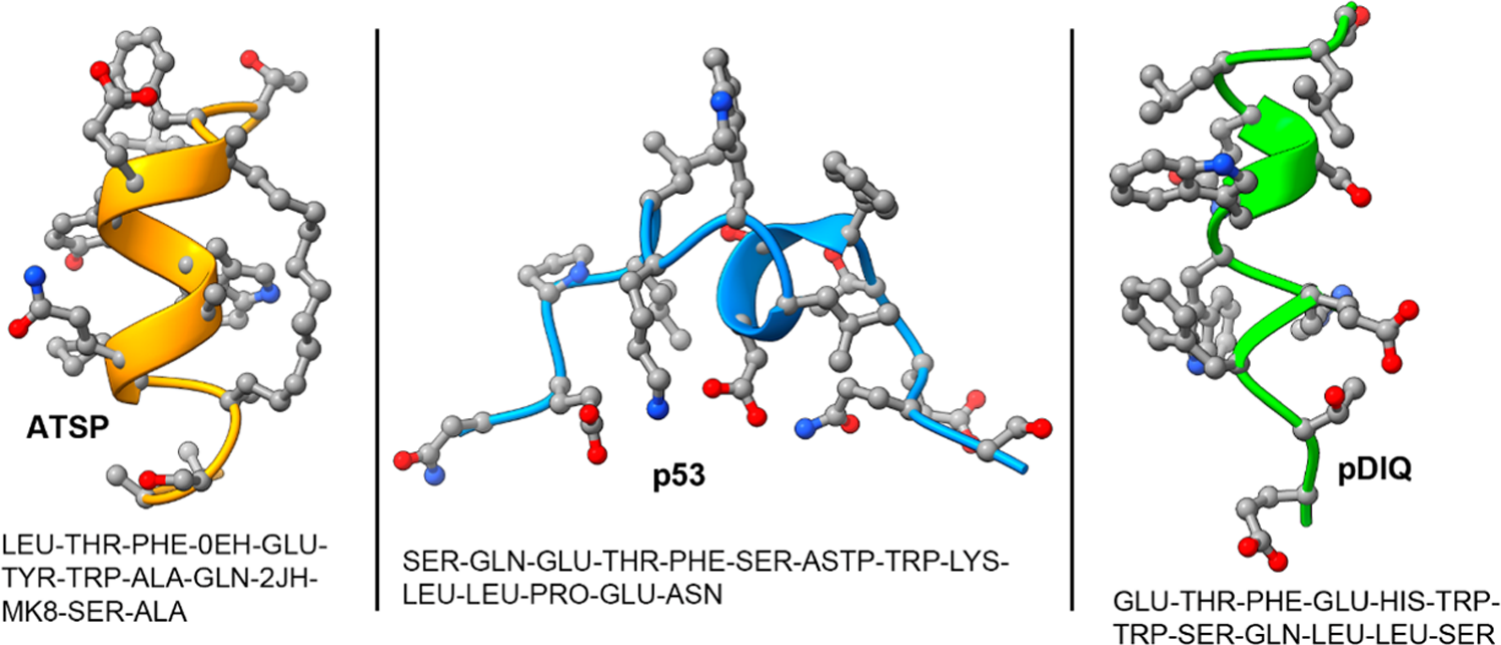
Peptide families used in this study. The peptide sequence is also indicated.

The physical nature of the interaction is based on two main factors. First, the polarizability of the tetrel atom (Tt), which increases upon descending in the group. Consequently, the electropositive region of the σ–hole increases if the EWG–Tt bond (EWG = electron-withdrawing group) is more polarized, resulting in a strengthening of the noncovalent interactions (NCI). Second, another way to polarize the EWG–Tt bond is by increasing the electron-withdrawing ability of EWG. Therefore, the combination of heavy elements and strong EWG increases the positive potential and size of the σ–hole, thus reinforcing the NCI (by increasing the contribution of electrostatics). In this regard, carbonyl groups typically exhibit a depletion of electrostatic potential above and below the molecular plane, which corresponds to the presence of a π-hole^29,30^ early manifested in the work of Burgi and Dunitz, and the proper spatial distribution of substituents to make it sterically accessible to a Lewis base. Since carbonyl groups are crucial components of protein and peptide backbones, we were interested in analyzing the impact of π-hole TtBs in the folding mechanism of p53, pDIQ, and ATSP peptides (see Figure 1).

To achieve that, we built on our previous success of identifying protein–peptide-bound states involving p53, pDIQ, and ATSP^31^ and performed a series of MELDxMD (modeling by employing limited data x molecular dDynamics) simulations using “free” p53, pDIQ, and ATSP peptides, which shed light on differences in their binding mechanisms. In addition, using quantum mechanics (QM) calculations at the B3LYP/def2-SVP level of theory, we identified several CO···CO TtBs that contribute to the stabilization of the backbone structure and quantifed them using Quantum Theory of Atoms in Molecules (QTAIM) and Noncovalent Interactions plot (NCIplot) analyses. In addition, we conducted a comparative study with the X-ray crystal structures of the three selected peptides to discuss the ability to sample these interactions during binding with current force fields (FF). Finally, using small model systems, we performed an ab initio study (RI-MP2/def2-TZVP level of theory), which allowed us to evaluate the strength of the interaction as well as the cooperativity between tetrel and hydrogen-bonding interactions.

We believe that characterizing and understanding these interactions is of general interest to understand the molecular recognition and aggregation phenomena since the finely tuned balance of energetic and entropic contributions in peptides results in small perturbations (or inaccuracies) that easily shift equilibrium distributions, resulting in the difference between accurate and erroneous predictions. We expect that the results gathered herein will be of interest to those scientists working in the field of computational biology (for instance, for accurate modeling with force field-based methods) as well as to structural biologists (to query and identify the prevalence of CO···CO TtBs in biological systems).

## Results and Discussion

### MD Simulations

The MDM2-p53 system has been widely simulated using MD approaches due to its biological relevance, especially in cancer. The p53 peptide epitope binds the homologous MDM2 and MDMX proteins by anchoring three hydrophobic residues in a hydrophobic cleft in either protein. For the three residues to satisfy the geometrical requirements of the cleft, the peptide adopts a helical conformation, whereas in its unbound form, it is intrinsically disordered. Though many peptides bind both proteins, the hydrophobic cleft is deeper in MDM2, leading to higher binding affinities. Peptide inhibitor design strategies have focused on either increasing the hydrophobic interactions (e.g., with non-natural amino acids) in the cleft or increasing the helicity of the peptide (e.g., through stapled peptides). Most simulation studies have focused on the p53-MDM2 interaction around the bound conformation or described the binding process of only p53 due to the computational expense of binding simulations. We have previously used an enhanced sampling approach (MELD, see Computational Methods) that combines force field preferences with guiding information through Bayesian inference to predict the structures and relative binding affinities of a series of peptides binding MDM2 and MDMX.^32,33^ The process samples multiple binding and unbinding events in which the peptides transition from unfolded states to helical states in the proximity of the binding site.^31^ As expected, peptides that had a higher degree of helicity (ATSP > PDIQ > p53) had higher populations of helical conformations in the replica exchange ensemble.

**Figure 2:**
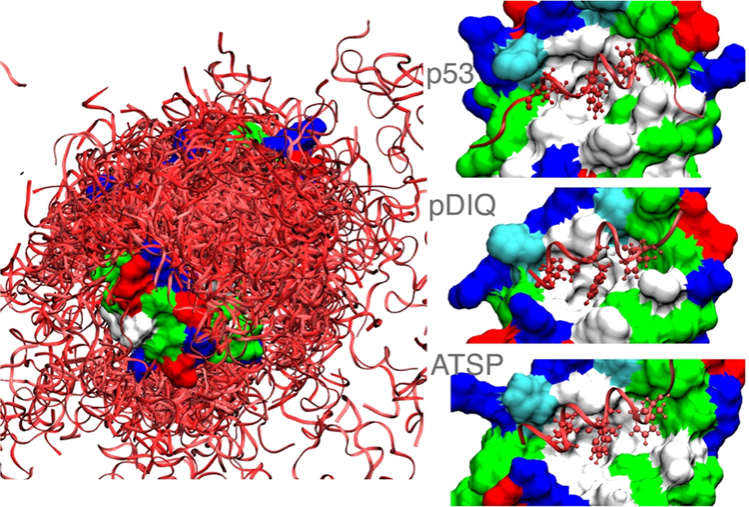
(Left) Low-temperature MELD ensemble of peptides (red ribbons) around the MDM2 protein (surface representation) with residue coloring scheme as follows: blue (charged positive), red (charged negative), green (polar), and white (hydrophobic). (Right) Centroid of the cluster representative of the native state (4th cluster for p53, 1st for pDIQ and ATSP). Side chains of the hydrophobic anchoring residues are represented explicitly.

We find that although force fields predict p53 as intrinsically disordered in its free form (largest cluster size below 1%), in agreement with experiments, it is of good quality to predict the folding of the peptide when it is in proximity to MDM2 (71% population for the highest cluster). We thus only use the information to favor multiple events in which the peptide is brought close to the protein binding site. Using the native contacts in the complex as guiding information trivially identifies the bound conformation but misses important details about the binding process. We found that creating a list of possible restraints between the hydrophobic anchoring residues in the peptide and the hydrophobic residues in the protein binding site favored a more extensive sampling of different peptide orientations, binding modes, and peptide conformations. Through the MELD approach, the peptide samples multiple binding/unbinding events, leading to (1) identification of the binding pocket and (2) recovering structures of the complex in good agreement with experimental structures (see Figure 2).

We simulated the binding of the native p53 peptide as well as two peptides with higher affinity (pDIQ and ATSP) (see Figure 2—a figure of the three peptides binding, their populations, and figure of the three peptides in solution and their populations). ATSP is a stapled peptide that adopts helical conformations in solution, while pDIQ is a designed peptide that has a large propensity toward helical conformations in its free form. Both pDIQ and ATSP are found preferentially in the binding site with the experimental binding conformations in the lowest temperature ensemble. However, for p53, the top three clusters exhibit one or two of the canonical anchoring residues in the binding site and additional hydrophobic residues (from the peptide) occupying this site. To satisfy these interactions, the peptide backbone structure adopts kinked conformations, unlike the experimental structure. It is the fourth population cluster in this case that predicts conformations where the anchoring side chains and helical structure match the experimental structure.

Previous simulations using a higher amount of information in MELD simulations recover the experimental p53 binding mode.^32,33^ Hence, we hypothesize that a small imbalance in the force field could account for the difference in conformational preferences. Even though the peptide in the presence of MDM2 adopts preferentially kinked conformations during the binding process, we found that only two-thirds of the time the peptide was binding in the active site when adopting this conformation. Thus, as the peptide moves from bulk solution to the vicinity of the protein, the conformational preferences shift to stabilize some preferred conformation with a high population. This conformation preferentially binds in the active site. Earlier work has described the prevalence of CO···CO TtB interactions in the stabilization of supramolecular interactions. These interactions have not typically been considered in force field developments and could thus be missing.

**Figure 3:**
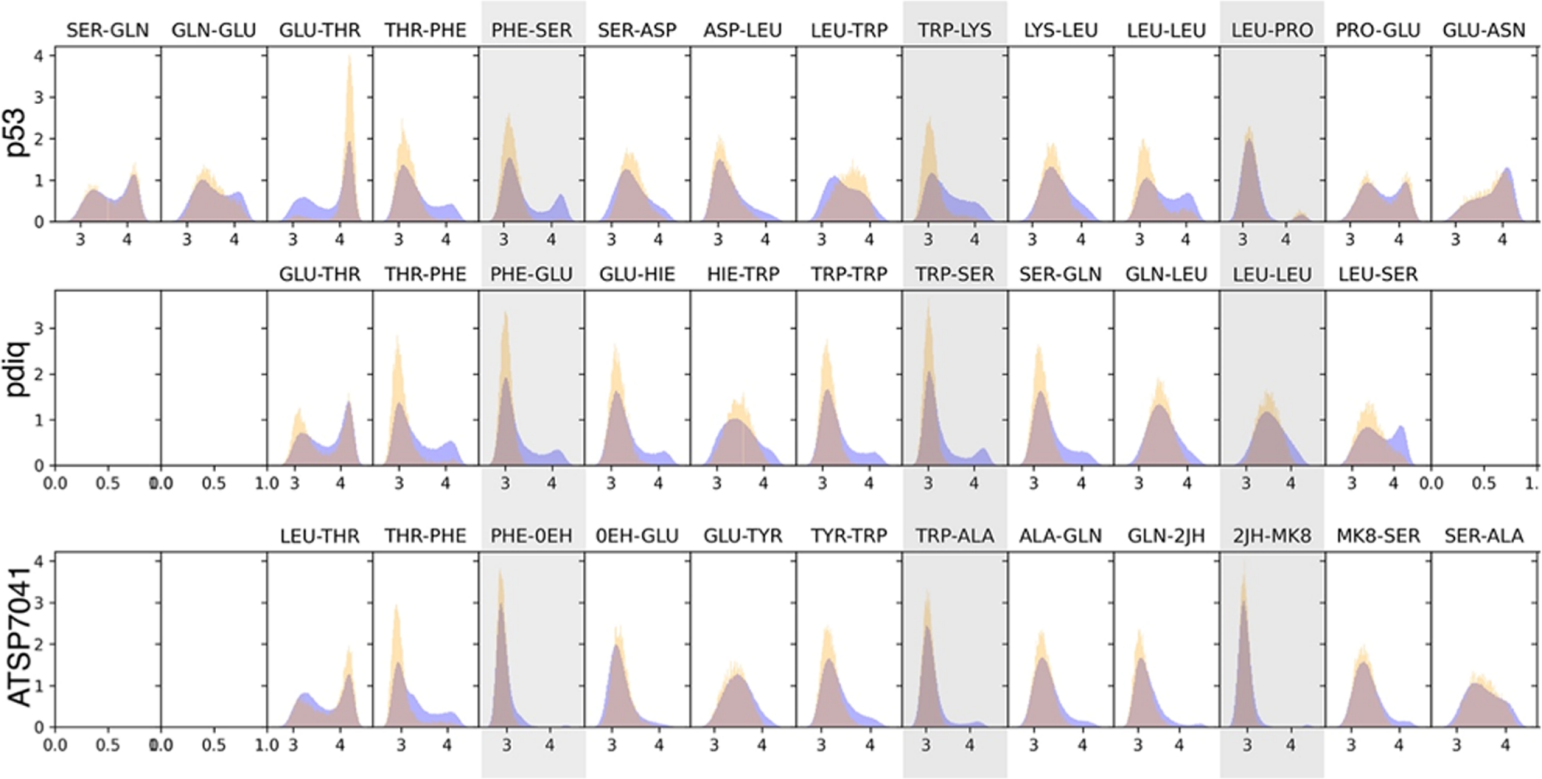
Distributions of CO···CO distances between consecutive residues in the peptide sequence for each peptide studied. The orange distribution represents the results considering only the lowest temperature replica ensemble. Blue distributions consider the ensemble based on the 30 replicas used in MELD calculations. Gray boxes denote the three hydrophobic residues that anchor on the MDM2 binding site.

We thus collected information about the distribution of CO···CO TtB interactions found in the MELD ensembles (see Figure 3). In the figure, we compare the distributions of the lowest replica ensemble (where simulations sample mostly bound or misbound states) and the ensemble produced by the 30 replicas (sampling both bound and unbound states). Here, the 30 replicas were not reweighted to account for their different temperatures. Instead, we just counted each frame with identical weight to assess the ability to sample different states for the three peptides during the binding process. As expected, we see broader distributions when using the 30 replica ensemble. Most importantly, the three anchoring residues present the most narrow distributions centered at short distances in the lowest temperature ensemble (except one anchoring Leucine in pDIQ). These distributions are narrowest for the well-structured ATSP peptide, followed by the pDIQ peptide and the p53 peptide. On the other hand, residues near either end of the peptide exhibit a broad distribution of distances for this type of interaction. To further understand the physical nature and extension in real space of the CO···CO that might be stabilizing peptide backbone conformations, we selected the three peptide conformations in the case of ATSP and pDIQ peptides and the top five conformations in the case of p53 peptide seen during binding simulations and then experimentally determined conformations for further QM analysis.

### QM Calculations

#### Computations Using Small Peptide Models

**Figure 4:**
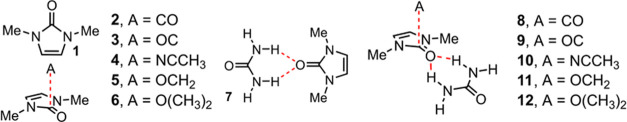
Compounds **1** and **7** and complexes **2** to **6** and **8** to **12** used in this study (A = Lewis base).

We started the QM study by performing *ab initio* calculations (RI-MP2/def2-TZVP level of theory) on a series of noncovalent complexes to study in detail the CO···CO TtB interaction. To achieve this, we used compound **1** (consisting of a cyclic amide) and compound **7** (composed of a bifurcated hydrogen bond (HB) complex between compound **1** and a urea molecule) to evaluate the synergistic effect between both TtB and HB interactions. In addition, we used CO, CH_3_CN, CH_2_O, and O(CH_3_)_2_ as electron-rich moieties (see Figure 4).

The results are gathered in Table 1, and as noted, the interaction energy obtained was attractive in all cases, ranging from weak (complexes **3** and **5**) to moderate (complexes **9** and **12**). Interestingly, complexes **8** to **12** achieved larger interaction energy values than complexes **2** to **6**, thus pointing to a reinforcement of the TtB due to the formation of a prior bifurcated HB complex. In addition, for CO involving complexes **2** and **3**, the former achieved a slightly larger interaction energy value (−1.7 and −1.4 kcal/mol, respectively), while the opposite behavior was observed for CO complexes **8** and **9** (−2.8 and −3.0 kcal/mol, respectively).

**Table 1:** BSSE-Corrected Interaction Energy Values (Δ*E*_BSSE_, in kcal/mol), Equilibrium Distances (*d*, in Å), and the Value of the Density at the Bond Critical Point That Characterizes the TtB (ρ × 100 TtB, in A.U.) and the Ancillary Interactions (ρ_Ancillary_ × 100) for Complexes **2** to **6** and **8** to **12**

complex	Δ*E*_BSSE_	*d*	ρ × 100 TtB	ρ_ancillary_ × 100
**2**	–1.7	3.144	0.63	0.60
**3**	–1.4	3.060	0.60	0.64
**4**	–1.9	3.076	0.69	0.74
**5**	–0.9	3.124	0.50^a^	0.56
**6**	–1.7	3.121	0.61	0.59
**8**	–2.8	3.136	0.66	0.56
**9**	–3.0	3.063	0.61	0.61
**10**	–2.5	3.033	0.76	0.74
**11**	–2.6	3.110	0.57^a^	0.50
**12**	–3.5	3.051	0.71	0.46

a In these complexes, the value of the density given was taken from the Noncovalent Interactions plot (NCIplot) isosurface located between the O and C atoms.

On the other hand, complex **4** involving CH_3_CN as an electron donor achieved a more favorable interaction energy value compared to complexes **2** and **3**; however, complex **10** was weaker than complexes **8** and **9** involving CO. Finally, complex **6** involving dimethylether achieved a comparable binding energy value to the CO involving complex **2** and a larger reinforcement of the TtB interaction upon establishment of a HB with the urea molecule (complex **12** = −3.5 kcal/mol and complex **8** = −2.8 kcal/mol). This will be discussed in the “atoms in molecules” (AIM) analysis (see below).

**Figure 5:**
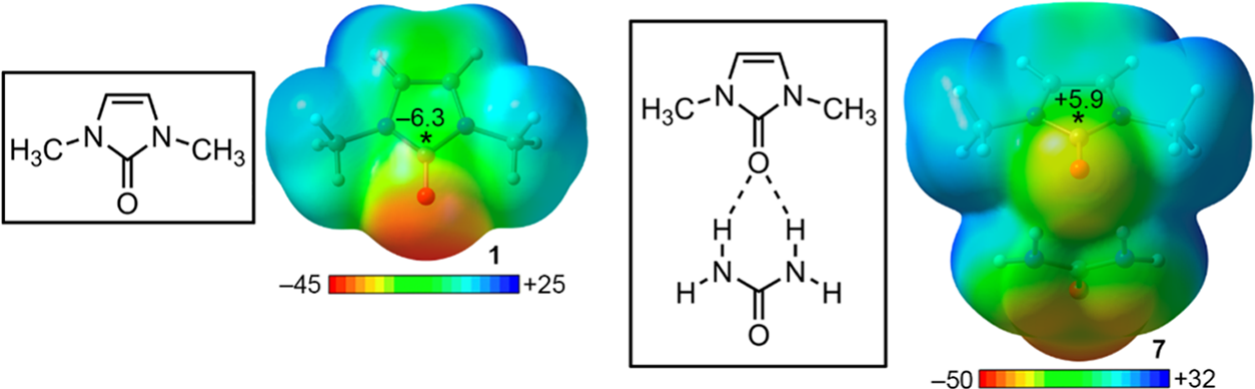
Electrostatic potential map of compounds **1** (left) and **7** (right). Energy values at concrete regions of the surface (*) are given in kcal/mol (0.001 a.u.).

To rationalize these findings, we computed the molecular electrostatic potential (MEP) surface of compounds **1** and **7** (see Figure 5). As noticed, in the case of compound **1**, the value of the electrostatic potential over the C atom belonging to the C=O bond is negative (−6.3 kcal/mol). Therefore, since electrostatics is not favorable in the case of complexes **2** to **6**, it is expected that other energy contributions (e.g., polarization and dispersion) as well as ancillary interactions (e.g., π–π stacking and CH−π) involving the π-system of compound **1** contributed to favorable interaction energy values obtained. On the contrary, in the case of compound **7**, the MEP value observed over the C atom from the carbonyl group was positive (+5.9 kcal/mol). This is due to the formation of a bifurcated HB with the urea molecule, which withdraws charge from the sp^2^ O atom. These results are useful to explain (from an electrostatic point of view) the reinforcement of the interaction energy observed from complexes **2**–**6** to complexes **8**–**12**.

**Figure 6:**
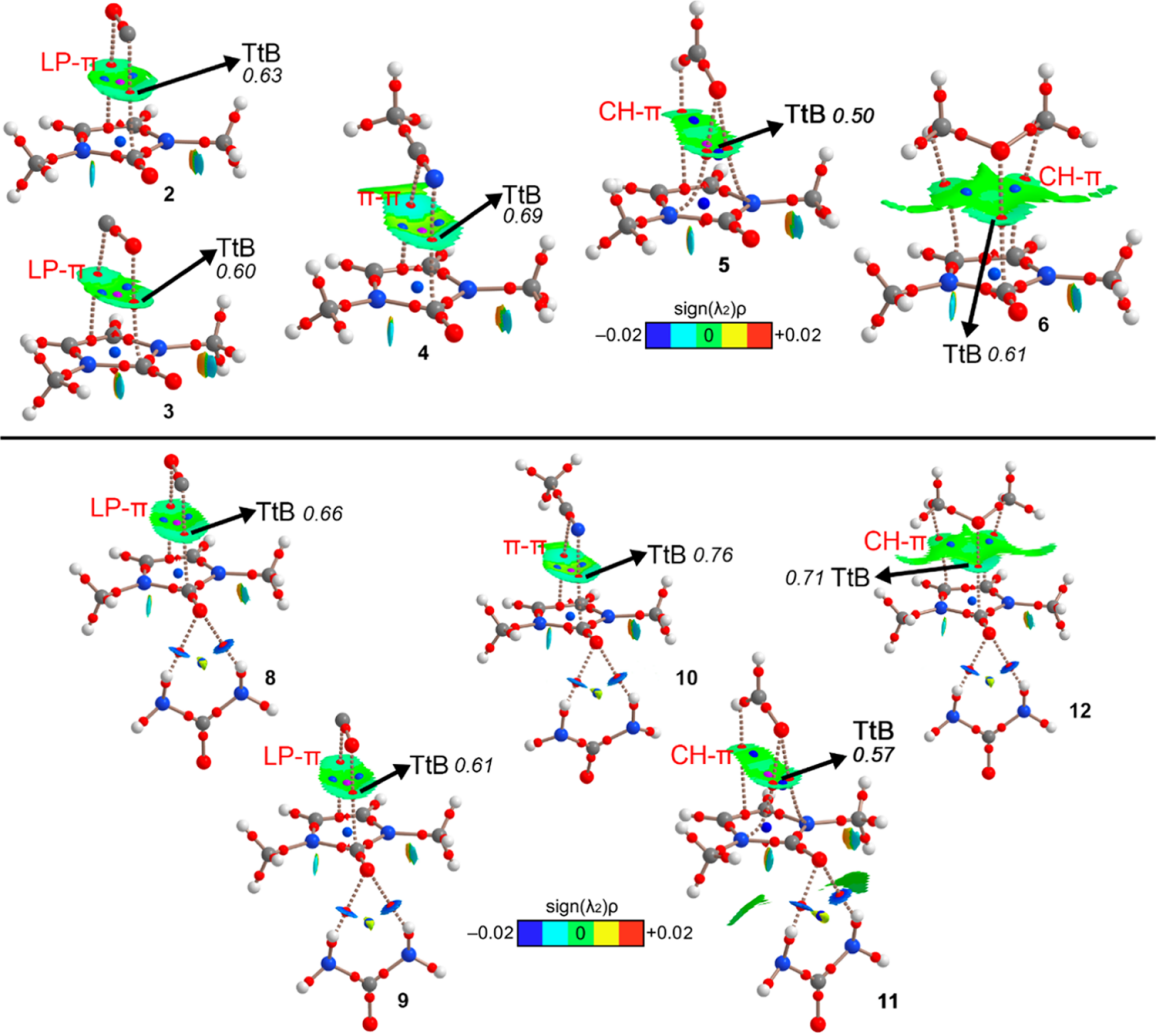
Distribution of critical points and bond paths for complexes **2** to **6** and **8** to **12** (bond, ring, and cage critical points are colored in red, blue, and magenta, respectively). The bond paths connecting bond critical points are also represented. The value of the density at the BCP that characterizes the TtB is given in a.u. Ancillary interactions are highlighted in red. NCIplot color range −0.002 a.u. ≤ (signλ_2_)ρ ≤ 0.002 a.u.

Also, to analyze the interaction from a charge-density perspective, we performed an AIM and NCIplot analyses of complexes **2** to **6** and **8** to **12** (see Figure 6). As noted, in all of the cases, the TtB interaction was characterized by the presence of a bond critical point (BCP) and a bond path connecting the C atom belonging to the carbonyl moiety of compound **1** and the electron donor atom (C, O, or N). In addition, ancillary interactions (LP−π in the case of complexes **2**, **3**, **8**, and **9**, π–π stacking in complexes **4** and **10**, and CH−π interactions in the case of complexes **5**, **6**, **11**, and **12**) were also denoted by the presence of one or two BCPs and bond paths connecting the π-system of the cyclic amide and the lone pairs, π-system, and CH bonds from the Lewis base. The presence of these ancillary interactions helps to rationalize the attractive interaction energy values obtained for compounds **2** to **6**, as mentioned above. Finally, in complexes **8** to **12**, two BCPs and bond paths connect the O atom from the carbonyl moiety to the two NH_2_ groups from the urea molecule, thus characterizing a bifurcated HB interaction.

Lastly, the NCIplot analyses of the TtB complexes studied herein showed a greenish isosurface located between the electron-rich molecule and the cyclic amide, which accounts for the presence and weak nature of the interaction. Also, the HBs established between compound **1** and the urea molecule exhibited a dark blue isosurface between the O and N–H groups, which denotes the presence of a strong and attractive interaction, as expected.

**Figure 7:**
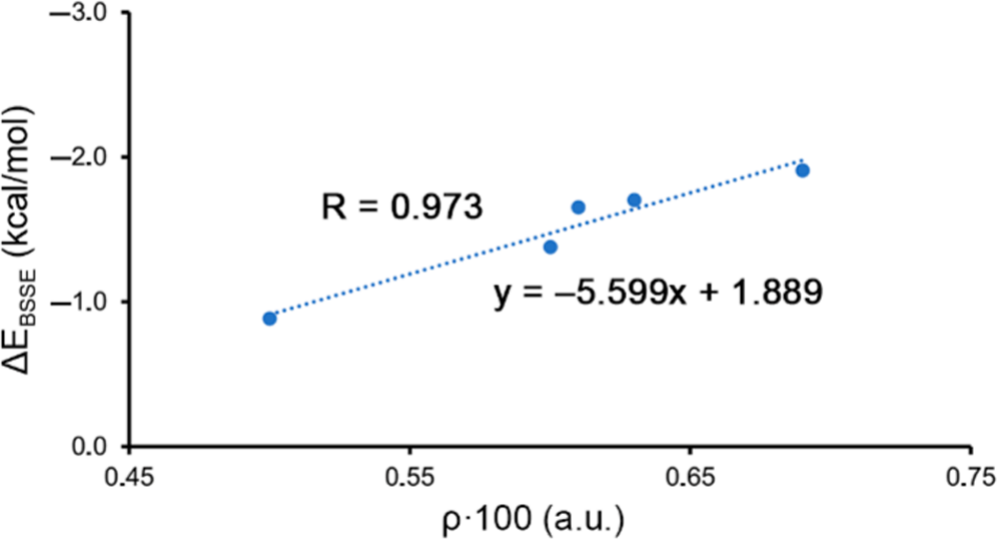
Regression plot for ρ × 100 (a.u.) values *vs* the interaction energies (Δ*E*_BSSE_) for TtB complexes **2** to **6**.

Lastly, with the purpose of transferring our observations in small models to the entire peptide structure, we carried out a graphic representation of the relationship between the MP2 BSSE-corrected interaction energies of complexes **2** to **6** and the value of the density that characterizes the TtB interaction (ρ × 100) (see Figure 7). As noticed, we obtained a very good agreement (*r* = 0.98), which remarks that the ρ values are good predictors of the TtB strength, in line with other noncovalent interactions.^34^ The presence of these additional contacts provokes an overestimation of the TtB strength if the value of ρ is used as TtB energy predictor by means of the equation of Figure 7. Since the values of the densities at the other intermolecular critical points (characterizing ancillary LP−π, π–π, and CH−π interactions) are similar to those of the tetrel bonds (see ρ_ancillary_ values in Table 1, last column), we have considered as a rough estimation that half of the energy is due to the TtB and the other half to the ancillary interactions. The only complex where the difference between ρ and ρ_ancillary_ is large is **12**, which is not used in the regression plot. Our next step was to interpolate the ρ × 100 values obtained from the intramolecular CO···CO TtB interactions exhibited by the three different peptides to estimate the strength of the interaction.

### Computations Using the Peptide Backbone

To provide evidence regarding the nature and strength of the CO···CO TtB studied herein, we built a theoretical model focusing on the peptide’s backbone, which allowed us to analyze the main noncovalent interactions (NCIs) responsible for the formation of the α helix motif.

### ATSP Peptide

**Figure 8:**
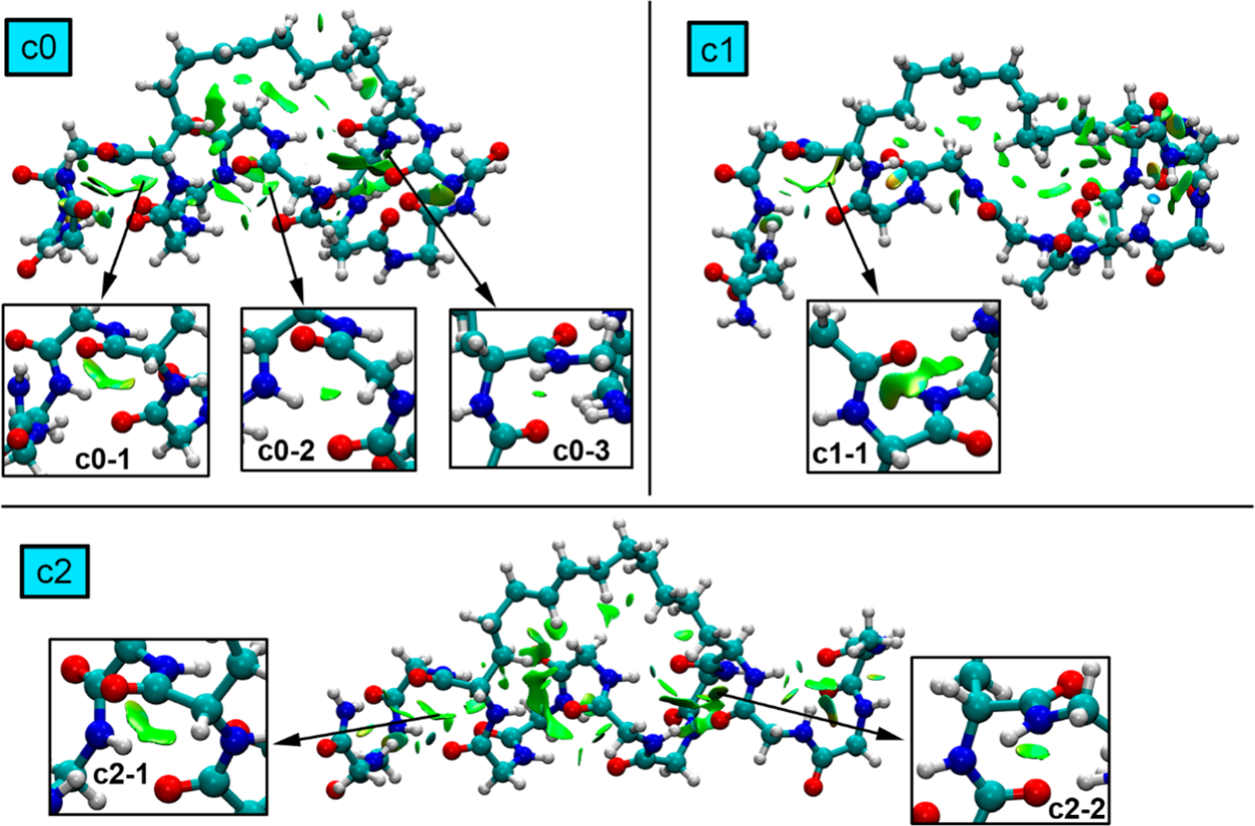
NCIplot surfaces for ATSP clusters **c0** to **c2**. The TtB interactions are magnified inside the square parts of the figure. The density and RDG cutoff values = 0.5 and 1.0, respectively. The density and RDG cutplot values = 0.07 and 0.3 a.u., respectively. Surfaces created using the fine multigrid option.

In Figure 8, we show the noncovalent interactions plot (NCIplot) analyses of the top three most populated clusters (**c0** to **c2**) related to ATSP peptide. As noticed in all three clusters, the peptide structure is stabilized by several CO···CO TtBs, which assist in the global stabilization of the ensemble. These are mainly located at opposite sides of the peptide chain in clusters **c0** (**c0**–**1** and **c0**–**3**) and **c2** (**c2**–**1** and **c2**–**2**), while in the case of **c1**, only one TtB interaction was found at one side of the peptide. Also, **c0** exhibits a TtB in the middle of the structure (**c0**–**2**).

For clusters **c0** and **c2**, two main CO···CO TtBs were found, involving the same residues in both clusters (**c0–1**/**c2–1**–2JH-MK8 and **c0–3**/**c2–2**–PHE-0EH). Interestingly, these two TtBs were the ones that presented the narrowest distance distributions in the MELD ensembles, thus indicating that TtBs participate in the most explored conformations along the course of the MELDxMD trajectory, likely acting as pincers that assist in holding the helical conformation in ATSP. Also, the carbonyl groups involved in the TtBs belong to the aromatic key residues for protein binding (e.g., PHE), thus remarking the importance of this interaction to stabilize the helical structure of the peptide when binding MDM2 protein. Besides, cluster c0 also presented another TtB interaction (**c0**–**2**), which was not carried over into cluster **c1**. This TtB involved residues TRP-ALA, which also exhibited a narrowed distribution along the course of the MELD trajectory. On the other hand, only one TtB was observed in cluster **c1** (2JH-MK8), likely pointing to HBs as the main driving force of this α helix conformation.

Interestingly, while the strength of the TtB established in all clusters seemed of similar magnitude (green isosurfaces were found in all cases), the computation of the intramolecular CO···CO interactions provided an estimation of the variation of its strength along these top 3 clusters from the MELDXMD trajectory. In this regard, the interaction energies regarding the TtBs present in clusters **c0** to **c2** are gathered in Table 2 using the equation shown in Figure 5. As noted, in all of the cases, attractive and moderately strong interaction energy values were obtained (ranging between −3.6 and −1.2 kcal/mol). In addition, among those clusters presenting two side CO···CO TtBs (**c0** and **c2**), the **c0**–**1** TtB exhibited a stronger TtB interaction than the **c0**–**3** TtB (−3.4 and −2.1 kcal/mol, respectively). However, the opposite was observed when comparing **c2** cluster TtBs (−2.3 kcal/mol for **c1**–**1** and −3.6 kcal/mol for **c2**–**2**), thus likely indicating that the stabilization effect of the TtBs varies along the MELDxMD trajectory at both sides of the peptide.

**Table 2:** Cluster ID, Peptide Residues Involved (resID), and the Value of the Density at the NCIplot Isosurface That Characterizes the TtB (ρ × 100), BSSE-Corrected Interaction Energy (Δ*E*_BSSE_, kcal/mol), and O···C Distance (*d*_Co···Co_, in Å) for ATSP Clusters **C0** to **C2**

cluster ID	resID	ρ × 100	Δ*E*_BSSE_	*d*_CO···CO_
**c0**–**1**	2JH···MK8	1.55	–3.4	2.710
**c0**–**2**	TRP···ALA	0.77	–1.2	3.092
**c0**–**3**	PHE···0EH	1.07	–2.1	2.927
**c1**–**1**	2JH···MK8	1.38	–2.9	2.792
**c2**–**1**	2JH···MK8	1.15	–2.3	2.875
**c2**–**2**	PHE···0EH	1.62	–3.6	2.704

In order to evaluate the sensitivity of the results to the method or basis set used, we run a short benchmarking on one of the clusters from ATSP peptide (c0) using B3LYP (with and without D3 dispersion correction) and M06–2X functionals in addition to def2-SVP and def2-TZVP basis sets. As noted in Table 3, the results were similar among the different levels of theory used. More in detail, **c0**–**1** and **c0**–**3** TtB energies remained almost identical when using D3 empirical dispersion correction, while **c0**–**2** increased 0.3 kcal/mol in strength. On the other hand, using a larger basis set (def2-TZVP) resulted in slightly more favorable interaction energy values (either using B3LYP or M06-2X functionals). Overall, the results were nonsensitive to the method, the basis set used, or the use of empirical dispersion correction parameters. In this regard, we recommend choosing a density functional theory (DFT) functional with proper treatment of electrostatics, since dispersion seems to have little impact on the strength of the TtB complexes studied herein.

**Table 3:** Level of Theory and BSSE-Corrected Interaction Energies (Δ*E*_BSSE_, kcal/mol) for the TtBs Present in ATSP Cluster **C0**–**1**, **C0**–**2**, and **C0**–**3**

level of theory	Δ*E*_BSSE_ (**c0**–**1**)	Δ*E*_BSSE_ (**c0**–**2**)	Δ*E*_BSSE_ (**c0**–**3**)
B3LYP/def2-SVP (this work)	–3.4	–1.2	–2.1
B3LYP-D3/def2-SVP	–3.4	–1.5	–2.0
B3LYP-D3/def2-TZVP	–3.5	–1.6	–2.1
M06-2X/def2-SVP	–3.4	–1.3	–2.1
M06-2X/def2-TZVP	–3.5	–1.6	–2.2

#### P53 Peptide

**Figure 9:**
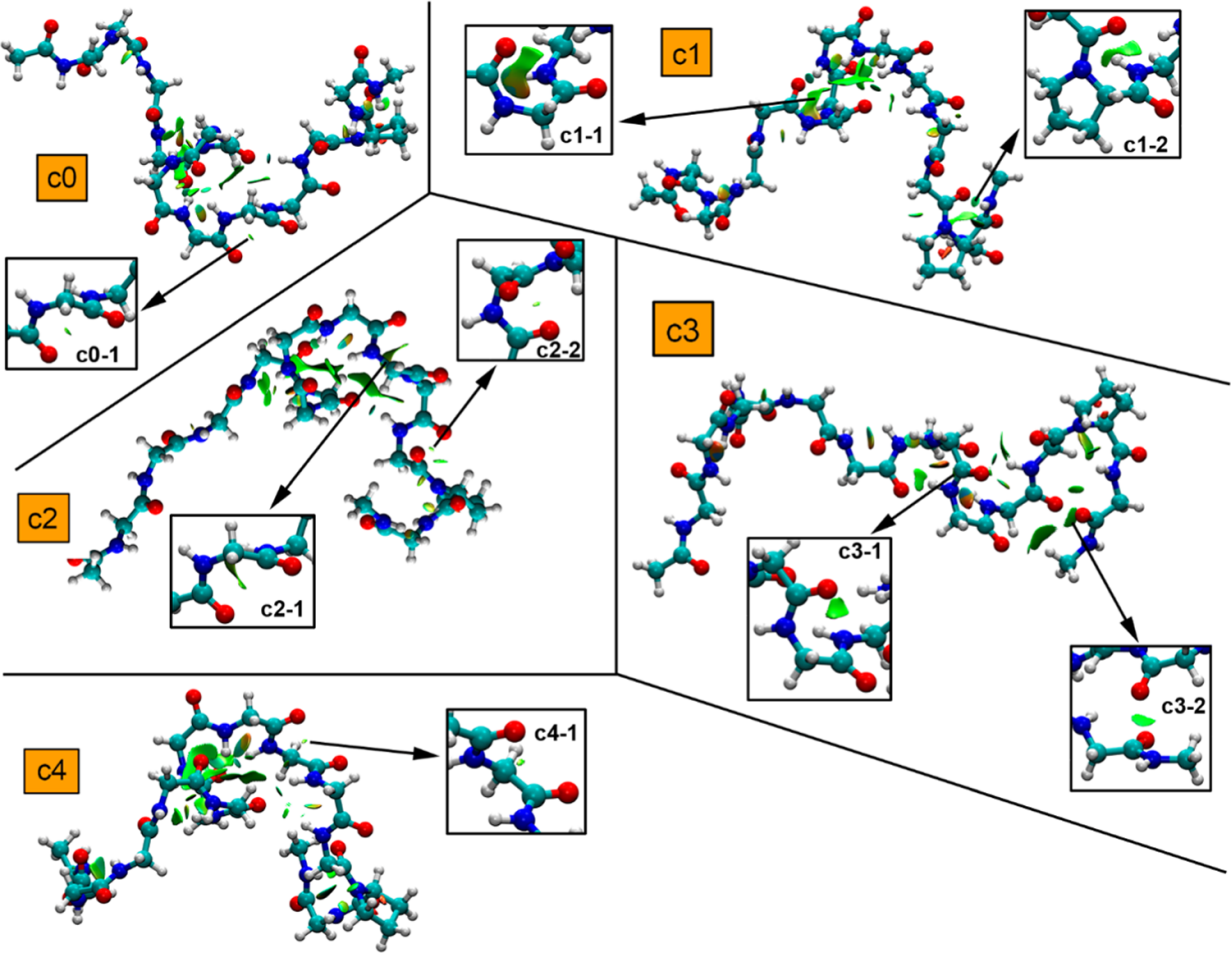
NCIplot surfaces for p53 clusters **c0** to **c4**. The TtB interactions are magnified inside the square parts of the figure. The density and RDG cutoff values = 0.5 and 1.0, respectively. The density and RDG cutplot values = 0.07 and 0.3 a.u., respectively. Surfaces created using the fine multigrid option.

In Figure 9, the NCIplot analyses of the top five most populated clusters (**c0** to **c4**) related to the p53 peptide are shown. As noted, in cluster **c0**, only one CO···CO TtB interaction was found involving residues TRP-LYS, while in the case of clusters **c1**, **c2**, and **c3**, two TtB interactions involving PHE-SER (**c1**–**1**), PRO-GLU (**c1**–**2**), LYS-LEU (**c2**–**1**), LEU-PRO (**c2**–**2**), TRP-LYS (**c3**–**1**), and LEU···ASN (**c3**–**2**) per cluster were observed. Finally, in cluster **c4**, only one TtB interaction was found involving LYS-LEU residues (**c4**–**1**). Interestingly, clusters **c0**, **c1**, and **c2** exhibited a larger number of TtBs, thus remarking the role of this interaction as a stabilization source over the course of the MELDxMD trajectory.

In Table 4, the interaction energies regarding the TtBs present in p53 clusters are gathered, ranging between −2.8 and −0.5 kcal/mol. These values are generally less favorable than those obtained for the ATSP peptide, in line with the longer intramolecular CO···CO distances observed (which account for a less propensity to undergo a helical conformation). Clusters **c0**–**1**, **c2**–**1**, **c2**–**2**, and **c4**–**1** exhibited the more favorable TtB energies and assisted in the stabilization and packing of the central and terminal parts of the peptide structure. On the other hand, clusters **c3**–**1** and **c3**–**2** presented the lowest interaction energy values of the set, as expected from their respective CO···CO distance values. These results are in line with the intrinsically disordered nature of the p53 peptide, which is less prone to fold in an α helix motif compared to the other two cases studied (ATSP and pDIQ).

**Table 4:** Cluster ID, Peptide Residues Involved (resID), the Value of the Density at the NCIplot Isosurface That Characterizes the TtB (ρ × 100), BSSE-Corrected Interaction Energy (Δ*E*_BSSE_, kcal/mol), and O···C Distance (*d*_Co···Co_, in Å) for P53 Clusters **C0** to **C4**

cluster ID	resID	ρ × 100	Δ*E*_BSSE_	*d*_CO···CO_
**c0**–**1**	TRP···LYS	1.32	–2.8	2.800
**c1**–**1**	PHE···SER	0.85	–1.5	2.997
**c1**–**2**	PRO···GLU	0.92	–1.7	3.060
**c2**–**1**	LYS···LEU	1.22	–2.5	2.829
**c2**–**2**	LEU···PRO	1.24	–2.5	2.835
**c3**–**1**	TRP···LYS	0.59	–0.7	3.357
**c3**–**2**	LEU···ASN	0.51	–0.5	3.376
**c4**–**1**	LYS···LEU	1.25	–2.6	2.821

### pDIQ Peptide

**Figure 10:**
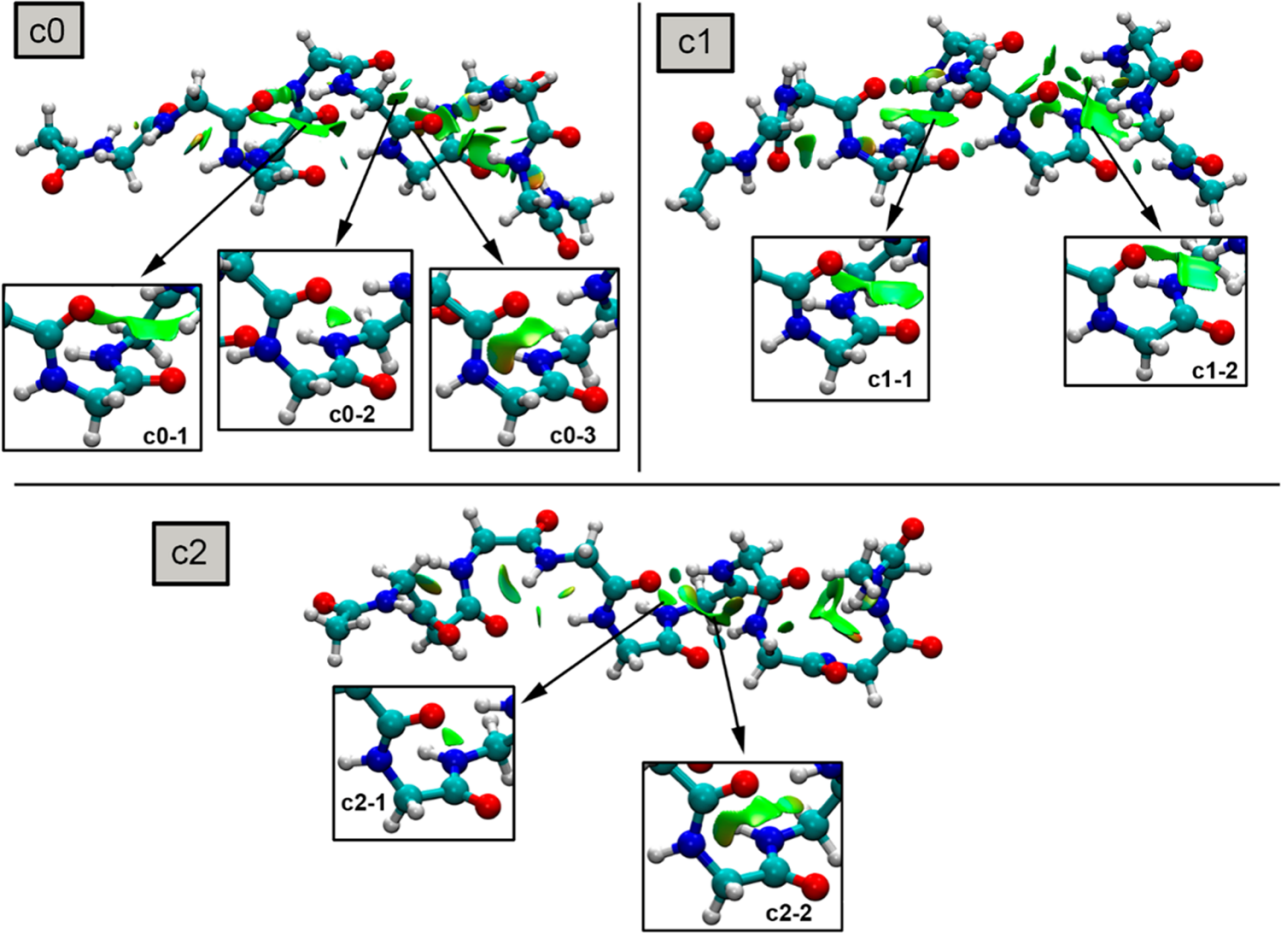
NCIplot surfaces for pDIQ clusters **c0** to **c2**. The TtB interactions are magnified inside the square parts of the figure. The density and RDG cutoff values = 0.5 and 1.0, respectively. The density and RDG cutplot values = 0.07 and 0.3 a.u., respectively. Surfaces created using the fine multigrid option.

In the case of pDIQ peptide (see Figure 10 and Table 5), the top three clusters exhibited at least two CO···CO TtBs along the MELDxMD trajectory (with **c0** exhibiting three TtBs); thus, O···C interactions play a noticeable role during the folding process regarding this peptide.

**Table 5:** Cluster ID, Peptide Residues Involved (resID), the Value of the Density at the NCIplot Isosurface That Characterizes the TtB (ρ × 100), BSSE-Corrected Interaction Energy (Δ*E*_BSSE_, kcal/mol), and O···C Distance (*d*_Co···Co_, in Å) for pDIQ Clusters **C0** to **C4**

cluster ID	resID	ρ × 100	Δ*E*_BSSE_	*d*^a^
**c0**–**1**	PHE···GLU	0.66	–0.9	3.126
**c0**–**2**	TRP···TRP	0.89	–1.6	2.943
**c0**–**3**	TRP···SER	1.34	–2.8	2.863
**c1**–**1**	PHE···GLU	0.97	–1.8	2.995
**c1**–**2**	TRP···SER	0.94	–1.7	2.993
**c2**–**1**	HIE···TRP	0.92	–1.7	2.989
**c2**–**2**	TRP···TRP	1.02	–1.9	2.943

In **c0**, three TtBs were observed (**c0**–**1** involving PHE-GLU), (**c0**–**2** involving TRP-TRP, and **c0**–**3** involving TRP-SER), as noted by the greenish isosurfaces located between the CO groups. Interestingly, two of them (**c0**–**1** and **c0**–**3**) involved PHE and TRP carbonyl groups, which achieved a narrow distance distribution in Figure 3 (see above), thus playing an important role in stabilizing the active peptide conformation upon binding to MDM2 protein. Among them, **c0**–**3** exhibited the most favorable TtB energy (in agreement with its shorter distance compared to **c0**–**1** and **c0**–**2** clusters), thus being the prominent TtB interaction.

In **c1**, the two TtBs observed implicated PHE-GLU residues (**c1**–**1**) and TRP-SER residues (**c1**–**2**); thus, these two TtBs were conserved among the two most populated clusters of the MELDxMD trajectory, remarking their importance since they involve two of the three aromatic key residues to achieve protein binding (PHE and TRP). In this case, both TtBs exhibited a similar strength (**c1**–**1** = −1.8 kcal/mol and **c1**–**2** = −1.7 kcal/mol), being of equal importance in the stabilization of the pDIQ α helical conformation. Finally, in **c2**, two TtB interactions were also found, involving HIE-TRP (**c2**–**1**) and TRP-TRP (**c2**–**2**). In this case, both TtBs were not related to the aromatic residues that participate in binding but still contributed to the overall stabilization of this cluster with −1.7 and −1.9 kcal/mol, respectively.

### Comparison with X-ray Peptide Structures

**Figure 11:**
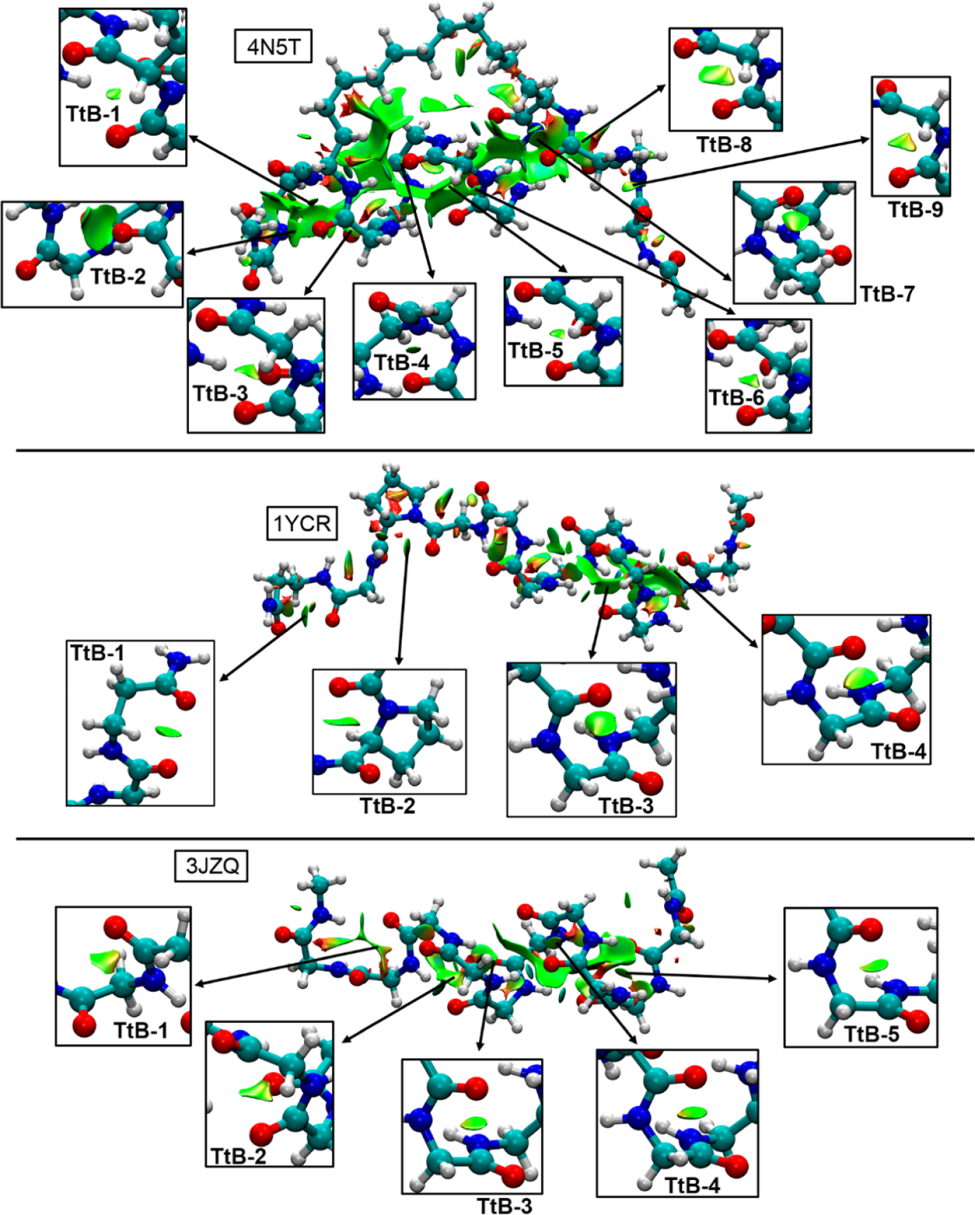
NCIplot surfaces for X-ray structures of peptides ATSP (top), p53 (middle), and Pdiq (bottom). The TtB interactions are magnified inside the square parts of the figure. The density and RDG cutoff values = 0.5 and 1.0, respectively. The density and RDG cutplot values = 0.07 and 0.5 a.u., respectively. Surfaces created using the fine multigrid option.

With the purpose of comparing the structures gathered from the clustering analysis to the experimental binding pose of the three selected peptide families, we carried out a similar analysis using PDBs 4N5T (ATSP), p53 (1YCR), and 3JZQ (pDIQ) (see Figure 11). It is important to note that these three backbone structures correspond to the folded conformation of each peptide on its bound state. Interestingly, we observed a larger number of O···C TtBs compared to those observed in the MELDxMD trajectories, that is, the number of stabilizing O···C TtBs increased from three to nine in ATSP, from one to four in p53, and from three to five in pDIQ peptide (taking **c0** as a reference in each case), in agreement with their respective propensity to form a helical structure. In Table 6, the interaction energies regarding the TtBs present in these X-ray structures are shown. As noticed, the magnitude of the TtBs was similar to that retrieved from the MD ensembles, thus indicating that the AMBER FF is able to efficiently sample through the conformational space of the three peptide families while capturing the binding process.

**Table 6:** Tetrel Bond ID (TtB ID) and the Value of the Density at the NCIplot Isosurface That Characterizes the TtB (ρ × 100), BSSE-Corrected Interaction Energy (Δ*E*_BSSE_, kcal/mol), and O···C Distance (*d*_Co···Co_, in Å) for ATSP (4N5T), P53 (1YCR), and pDIQ (3JZQ) X-ray Crystal Structures

4N5T			
TtB ID	ρ × 100	Δ*E*_BSSE_	*d*^a^
**1**	1.01	–1.9	3.008
**2**	0.56	–0.6	3.273
**3**	1.08	–2.1	2.960
**4**	1.05	–2.0	2.997
**5**	1.02	–1.9	2.977
**6**	1.04	–2.0	2.971
**7**	1.16	–2.3	2.909
**8**	1.24	–2.5	2.841
**9**	1.29	–2.7	2.828

1YCR			
TtB ID	ρ × 100	Δ*E*_BSSE_	*d*^a^
**1**	0.76	–1.2	3.585
**2**	1.21	–2.5	2.885
**3**	0.97	–1.8	3.011
**4**	1.32	–2.8	2.830

3JZQ			
TtB ID	ρ × 100	Δ*E*_BSSE_	*d*^a^
**1**	1.06	–2.0	2.855
**2**	1.15	–2.3	2.947
**3**	0.88	–1.5	3.025
**4**	1.14	–2.5	2.869
**5**	1.10	–2.2	2.892

## Conclusions

Proteins and peptides exhibit an exquisite balance between entropic driving forces that favor disordered states and stabilizing energetic contributions that favor well-defined structures. Most proteins lie at the edge of stability, with a net stabilization energy of just a few hydrogen bonds. It is surprising that fixed-charge force field models are able to reproduce some of the details that lead to stable structures and folding routes. Here, we find that when driving forces favor one particular state (pDIQ and ATPS), the potential of the binding partner is enough to drive binding to native-like conformations. For systems that lie at the edge of stability, such as the p53-MDM2 system, the force field is enough to sample native-like states in high populations but not the highest population cluster. We thus looked at the presence of a particular type of interactions that has been recently described in the literature. The presence of the interaction in the different states studied cannot indicate that it is a driving force during the folding process; it is nonetheless interesting to increase our awareness of nontraditional interactions involved in supramolecular recognition. Additionally, QM calculations shed light on the nature and number of stabilizing O···C tetrel bonds, being a complementary source of stability during the α helix formation process of the three selected peptide families. Particularly, a short computational study using a cyclic peptide model allowed us to provide new insights into the strength, directionality, and synergistic nature of TtB and HB interactions present in these systems. On the other hand, the combination of AIM and NCIplot techniques was useful to decipher the number and relative strength of TtB interactions on the top three (for ATSP and pDIQ peptides) and top five (in the case of p53 peptide) most populated clusters gathered from the MELDxMD trajectory. Finally, we provided an estimation of the strength of these intramolecular TtBs, which exhibited values that range from weak to moderately strong, with a dependence on the peptide family and cluster population. We believe the results from this study will be useful for those scientists working in the fields of rational drug design, structural biology, and computational chemistry as well as for expanding the relevance of tetrel bonds among the biological community.

## Computational Methods

### Molecular Dynamics Simulations

All simulations were carried out using the ff14SB force field^35^ for side chains and ff99SB force field^36^ for backbone parameters coupled with the GBneck2 implicit solvent model (igb = 8).^37^ Simulations of the free peptides were performed using the Amber package, and MELDxMD binding simulations were performed using the OpenMM package with the MELD plugin.^38^ MELDxMD simulations used 30 replicas with a one-dimensional H,T-REMD ladder following previous work.^32^ Cpptraj was used to analyze the ensembles. Clustering was performed by using a hierarchical model with epsilon set to two and using a *C*_α_ and *C*_β_ RMSD as a similarity measure. Clustering was done either on the peptide alone (to identify helical content found in the ensemble) or on the protein–peptide complex to identify bound conformations.

### Quantum Mechanics Calculations

#### Complexes **2** to **6** and **8** to **12**

The binding energies of complexes **2** to **6** and **8** to **12** were computed at the RI-MP2^39^/def2-TZVP^40^ level of theory. This level of theory has been demonstrated to be appropriate for the study of σ-hole interactions involving neutral and charged electron donors.^41^ The calculations have been performed by using the program TURBOMOLE version 7.0.^42^ The Cs symmetry point group was imposed during the optimization procedure. The binding energies were obtained using the supermolecule approximation, where Δ*E*_complex_ = Δ*E*_monomer1_ – Δ*E*_monomer2_. The results were corrected for the basis set superposition error following the Boys and Bernardi counterpoise method.^43^ In addition, calculations for the molecular electrostatic potential (MEP) surfaces and wave function analysis have been carried out using Gaussian 16 software.^44^ The Bader’s ″atoms in molecules″ theory^45^ has been used to study the interactions discussed herein by means of the AIMall calculation package.^46^ The wave function analysis has been carried out at the MP2/def2-TZVP level of theory.

Regarding the choice of the computational models, we chose a cyclic amide as a theoretical model of a peptide to impose Cs symmetry with the purpose of obtaining an energy minimum based on a single CO···CO tetrel bond presenting the minimum number of ancillary interactions. On the other hand, the use of a urea molecule as a molecular model allowed us to solely evaluate the contribution of having a pre-established bifurcated H-bond on the strength of the CO···CO tetrel bond, without additional interactions involved.

#### Clusters C0 to C4 of ATSP, P53, and PDIQ Peptides

We used force field-derived structures; therefore, no geometry optimization was performed before the calculation of the wave function (B3LYP^47,48^/def2-SVP level of theory). The NCIplot^49,50^ index allows convenient visualization of both inter and intramolecular interactions in real space. It plots isosurfaces of the reduced density gradient (related to |∇|/ρ^4/3^), which are colored in agreement with values of the electron density. The NCI contacts are characterized by the regions of small reduced density gradient (RDG) at low densities, being mapped in real space by plotting an isosurface of *s* for a low value of RDG. Besides, the sign of the second eigenvalue of the density Hessian times the density is color-mapped onto the isosurfaces, which allows the characterization of both the strength and (un)favorable nature of the interactions. More precisely, the color scheme is composed of a red–yellow–green–blue scale using red for repulsive (ρ_cut_^+^) and blue for attractive (ρ_cut_^–^) NCI interaction density. Weak repulsive and weak attractive interactions are identified by yellow and green surfaces, respectively.

## Data Availability

Protein Data Bank (https://www.rcsb.org/), Turbomole software (https://www.turbomole.org/), Gaussian 16 (https://gaussian.com/gaussian16/), AIMall (http://aim.tkgristmill.com/), NCIplot (https://www.lct.jussieu.fr/pagesperso/contrera/index-nci.html), VMD (https://www.ks.uiuc.edu/Research/vmd/), MELDxMD (http://meldmd.org/). All data needed to reproduce both MD and QM calculations can be found at https://zenodo.org/badge/latestdoi/587021643 and https://github.com/PDNALab/QM_MM_Study_P53-MDM2_Binding.git.

## Supporting Information Available

Cartesian coordinate complexes **2** to **6** and **8** to **12** as well as the backbone structures used for ATSP, p53, and pDIQ peptides (PDF)
