# Supplementary material for: Computational Study of Driving Forces in ATSP, PDIQ, and P53 Peptide Binding: C=O···C=O Tetrel Bonding Interactions at Work
Source: J Chem Inf Model. 2023 Apr 4;63(10):3018–29. doi: 10.1021/acs.jcim.3c00024 (PMC10207270; doi:10.1021/acs.jcim.3c00024)
Supplement: Supplementary file 1 — ci3c00024_si_001.pdf [file ci3c00024_si_001.pdf]

Supporting Information

Computational study of driving forces in ATSP, PDIQ and p53 peptide binding: C=O···C=O tetrel bonding interactions at work

Lijun Lang,[a] Antonio Frontera,[b] Alberto Pérez*,[a] Antonio Bauzá*,[b]

[a]Chemistry Department, University of Florida, Gainesville, FL 32611, USA; E-mail: perez@chem.ufl.edu; Tel.: +1-352 3927009
[b]Department of Chemistry, Universitat de les Illes Balears, Crta. de Valldemossa km 7.5, 07122 Palma Baleares), SPAIN; Fax: +) 34 971 173426; E-mail: antonio.bauza@uib.es

Cartesian coordinates of complexes 2 to 6 and 8 to 12 Page 2
Cartesian coordinates of peptide backbones Page 6

Cartesian coordinates of complexes to 2 to 6 and 8 to 12

2.

C	0.0303736	1.3653433	0.6791711
C	0.0303736	1.3653433	-0.6791711
H	-0.1649943	2.1629735	1.3758827
H	-0.1649943	2.1629735	-1.3758827
N	0.3288543	0.0807407	-1.0921060
N	0.3288543	0.0807407	1.0921060
C	0.5425928	-0.7449329	0.0000000
O	0.8559120	-1.9312473	0.0000000
C	0.4569972	-0.3883215	-2.4511992
H	0.6448086	-1.4596868	-2.4029444
H	-0.4626859	-0.2054431	-3.0085090
H	1.2909067	0.1018197	-2.9562398
C	0.4569972	-0.3883215	2.4511992
H	-0.4626859	-0.2054431	3.0085090
H	0.6448086	-1.4596868	2.4029444
H	1.2909067	0.1018197	2.9562398
O	-3.0470094	0.2040637	0.0000000
C	-2.6000158	-0.8427353	0.0000000

3.

C	0.1309287	1.3665743	0.6793562
C	0.1309287	1.3665743	-0.6793562
H	-0.0066758	2.1760566	1.3762237
H	-0.0066758	2.1760566	-1.3762237
N	0.3457501	0.0656791	-1.0918456
N	0.3457501	0.0656791	1.0918456
C	0.4889785	-0.7737093	0.0000000
O	0.6927668	-1.9844477	0.0000000
C	0.4161111	-0.4146668	-2.4511265
H	0.5775948	-1.4902807	-2.4017025
H	-0.5161295	-0.2124458	-2.9806069
H	1.2452018	0.0516389	-2.9851712
C	0.4161111	-0.4146668	2.4511265
H	-0.5161295	-0.2124458	2.9806069
H	0.5775948	-1.4902807	2.4017025
H	1.2452018	0.0516389	2.9851712
C	-2.9973378	0.3638878	0.0000000
O	-2.5699698	-0.6908423	0.0000000

4.

C	0.3148328	1.2266861	0.6805082
C	0.3148328	1.2266861	-0.6805082
H	-0.1079846	1.9318718	1.3770238
H	-0.1079846	1.9318718	-1.3770238
N	1.0588107	0.1407782	-1.0927028
N	1.0588107	0.1407782	1.0927028
C	1.5262459	-0.5722320	0.0000000
O	2.2110731	-1.5879933	0.0000000
C	1.2239323	-0.3367234	-2.4437990
H	1.9761521	-1.1232692	-2.4174936
H	0.2897182	-0.7534559	-2.8258389
H	1.5607238	0.4709540	-3.0943394
C	1.2239323	-0.3367234	2.4437990
H	0.2897182	-0.7534559	2.8258389
H	1.9761521	-1.1232692	2.4174936
H	1.5607238	0.4709540	3.0943394
N	-1.3138425	-1.7543417	0.0000000
C	-2.0796639	-0.8694686	0.0000000
C	-3.0582704	0.2092951	0.0000000
H	-2.9248656	0.8309805	0.8844331
H	-2.9248656	0.8309805	-0.8844331
H	-4.0681816	-0.2009036	0.0000000

5.

C	0.2122195	1.3535283	0.6796552
C	0.2122195	1.3535283	-0.6796552
H	-0.0953857	2.1153110	1.3765082
H	-0.0953857	2.1153110	-1.3765082
N	0.7655628	0.1576151	-1.0921030
N	0.7655628	0.1576151	1.0921030
C	1.0970011	-0.6254057	0.0000000
O	1.5814372	-1.7526609	0.0000000
O	-1.9847532	-1.1363832	0.0000000
C	-2.8985759	-0.3407653	0.0000000
H	-2.7144287	0.7462083	0.0000000
H	-3.9523780	-0.6687889	0.0000000
C	0.8639360	-0.3320288	2.4447927
H	-0.1244586	-0.5516707	2.8536081
H	1.4460117	-1.2514600	2.4125195
H	1.3679631	0.3976031	3.0793953
C	0.8639360	-0.3320288	-2.4447927
H	1.4460117	-1.2514600	-2.4125195
H	-0.1244586	-0.5516707	-2.8536081
H	1.3679631	0.3976031	-3.0793953

6.

C	1.4224390	-0.8916572	0.6794867
C	1.4224390	-0.8916572	-0.6794867
H	2.2171157	-0.6832504	1.3761175
H	2.2171157	-0.6832504	-1.3761175
N	0.1497650	-1.2345299	-1.0918494
N	0.1497650	-1.2345299	1.0918494
C	-0.6711015	-1.4582986	0.0000000
O	-1.8516713	-1.7926779	0.0000000
C	-0.3156301	-1.3809959	-2.4484040
H	-1.3977120	-1.4960825	-2.4061310
H	-0.0655874	-0.4960852	-3.0349196
H	0.1199222	-2.2625019	-2.9230449
C	-0.3156301	-1.3809959	2.4484040
H	-0.0655874	-0.4960852	3.0349196
H	-1.3977120	-1.4960825	2.4061310
H	0.1199222	-2.2625019	2.9230449
O	-0.8086327	1.6593686	0.0000000
C	-0.2257695	2.2060678	-1.1601817
H	0.8466112	1.9791428	-1.2093349
H	-0.7290552	1.7605075	-2.0167035
H	-0.3563962	3.2951888	-1.1962945
C	-0.2257695	2.2060678	1.1601817
H	-0.7290552	1.7605075	2.0167035
H	0.8466112	1.9791428	1.2093349
H	-0.3563962	3.2951888	1.1962945

8.

C	1.0478330	1.8580362	0.6792576
C	1.0478330	1.8580362	-0.6792576
H	1.6579406	2.4036797	1.3787362
H	1.6579406	2.4036797	-1.3787362
N	0.0533259	0.9922386	-1.0891845
N	0.0533259	0.9922386	1.0891845
C	-0.5605495	0.4181954	0.0000000
O	-1.4585336	-0.4385049	0.0000000
C	-0.2820593	0.6496555	-2.4526608
H	-1.2696457	0.1912411	-2.4465780
H	-0.3084584	1.5527710	-3.0619410
H	0.4423505	-0.0539580	-2.8646589
C	-0.2820593	0.6496555	2.4526608
H	-0.3084584	1.5527710	3.0619410
H	-1.2696457	0.1912411	2.4465780
H	0.4423505	-0.0539580	2.8646589
O	-1.4502929	4.0153931	0.0000000

C	-2.1529570	3.1200592	0.0000000
C	0.7939010	-3.1124495	0.0000000
N	0.3349328	-2.5134691	-1.1552676
H	-0.5235986	-1.9799143	-1.0920035
H	0.4666286	-3.0826229	-1.9761113
N	0.3349328	-2.5134691	1.1552676
H	0.4666286	-3.0826229	1.9761113
H	-0.5235986	-1.9799143	1.0920035
O	1.5899333	-4.0380088	0.0000000

9.

C	1.5233230	1.5118587	0.6794593
C	1.5233230	1.5118587	-0.6794593
H	2.2564799	1.8761429	1.3787766
H	2.2564799	1.8761429	-1.3787766
N	0.3358455	0.9392989	-1.0889061
N	0.3358455	0.9392989	1.0889061
C	-0.4106737	0.5562025	0.0000000
O	-1.5146489	-0.0127438	0.0000000
C	-0.0823411	0.7064594	-2.4525881
H	-1.1563651	0.5274471	-2.4446549
H	0.1309145	1.5875262	-3.0573318
H	0.4270904	-0.1623039	-2.8711023
C	-0.0823411	0.7064594	2.4525881
H	0.1309145	1.5875262	3.0573318
H	-1.1563651	0.5274471	2.4446549
H	0.4270904	-0.1623039	2.8711023
C	-0.0938403	-3.2089197	0.0000000
N	-0.3684900	-2.5060378	-1.1552865
H	-1.0453913	-1.7553889	-1.0922251
H	-0.3994154	-3.0892194	-1.9762749
N	-0.3684900	-2.5060378	1.1552865
H	-0.3994154	-3.0892194	1.9762749
H	-1.0453913	-1.7553889	1.0922251
O	0.4139309	-4.3190088	0.0000000
O	-1.2617696	3.4989944	0.0000000
C	-0.3762990	4.2139087	0.0000000

10.

C	0.9680567	1.6680408	0.6802821
C	0.9680567	1.6680408	-0.6802821
H	1.5710237	2.2229917	1.3793846
H	1.5710237	2.2229917	-1.3793846
N	0.0276830	0.7463447	-1.0897281
N	0.0276830	0.7463447	1.0897281
C	-0.5671990	0.1504703	0.0000000
O	-1.4239061	-0.7426614	0.0000000
C	-0.3606648	0.4544446	-2.4498381
H	-1.2553334	-0.1646412	-2.4081809
H	-0.5932590	1.3789081	-2.9788980
H	0.4312212	-0.0799037	-2.9766303
C	-0.3606648	0.4544446	2.4498381
H	-0.5932590	1.3789081	2.9788980
H	-1.2553334	-0.1646412	2.4081809
H	0.4312212	-0.0799037	2.9766303
C	1.1367588	-3.0465218	0.0000000
N	0.4130197	-2.8384791	-1.1493748
H	-0.4194758	-2.2667037	-1.1046325
H	0.9541264	-2.8444673	-1.9968241
N	0.4130197	-2.8384791	1.1493748
H	0.9541264	-2.8444673	1.9968241
H	-0.4194758	-2.2667037	1.1046325
O	2.2934601	-3.4442249	0.0000000
N	-2.3529270	2.6299611	0.0000000
C	-1.6128954	3.5334027	0.0000000
H	-0.9460869	4.3665042	0.0000000

11.

C	-1.2698753	1.4271654	0.6797803
C	-1.2698753	1.4271654	-0.6797803
H	-1.6826231	2.1338770	1.3797303
H	-1.6826231	2.1338770	-1.3797303
N	-0.5806622	0.3044146	-1.0885336
N	-0.5806622	0.3044146	1.0885336
C	-0.1379545	-0.4061918	0.0000000
O	0.5285670	-1.4540054	0.0000000
O	-3.0976320	-1.3630628	0.0000000
C	-4.1303631	-0.7292226	0.0000000
H	-4.1287114	0.3734469	0.0000000
H	-5.1155174	-1.2259304	0.0000000
C	-0.3452387	-0.1162628	2.4494723
H	-1.2598850	-0.0075586	3.0321019
H	-0.0602029	-1.1668918	2.4254006
H	0.4557459	0.4673763	2.9052923
C	-0.3452387	-0.1162628	-2.4494723
H	-0.0602029	-1.1668918	-2.4254006
H	-1.2598850	-0.0075586	-3.0321019
H	0.4557459	0.4673763	-2.9052923
C	3.6094756	0.1811033	0.0000000
O	4.6920910	0.7466795	0.0000000
N	2.9224258	-0.1306575	1.1545599
N	2.9224258	-0.1306575	-1.1545599
H	3.5044166	-0.1288582	1.9766975
H	2.2059213	-0.8440128	1.0899072
H	3.5044166	-0.1288582	-1.9766975
H	2.2059213	-0.8440128	-1.0899072

12.

C	1.7108645	0.5487520	0.6795843
C	1.7108645	0.5487520	-0.6795843
H	2.4599884	0.8790557	1.3790858
H	2.4599884	0.8790557	-1.3790858
N	0.5098689	0.0061576	-1.0886556
N	0.5098689	0.0061576	1.0886556
C	-0.2500275	-0.3460216	0.0000000
O	-1.3699747	-0.8821010	0.0000000
C	0.0812211	-0.2156824	-2.4493631
H	0.5021794	-1.1409991	-2.8457734
H	-1.0059142	-0.2838996	-2.4512358
H	0.3910910	0.6237922	-3.0712895
C	0.0812211	-0.2156824	2.4493631
H	-1.0059142	-0.2838996	2.4512358
H	0.5021794	-1.1409991	2.8457734
H	0.3910910	0.6237922	3.0712895
C	-0.2068981	-4.1675477	0.0000000
N	-0.4096566	-3.4403134	-1.1546241
H	-1.0069508	-2.6244943	-1.0882674
H	-0.5000106	-4.0166724	-1.9759019
N	-0.4096566	-3.4403134	1.1546241
H	-0.5000106	-4.0166724	1.9759019
H	-1.0069508	-2.6244943	1.0882674
O	0.1855968	-5.3240134	0.0000000
O	-0.8949675	2.6363639	0.0000000
C	-0.4497521	3.3003296	-1.1606241
H	0.6463032	3.3228424	-1.2081726
H	-0.8372184	2.7517033	-2.0177054
H	-0.8238782	4.3310882	-1.1982897
C	-0.4497521	3.3003296	1.1606241
H	-0.8238782	4.3310882	1.1982897
H	-0.8372184	2.7517033	2.0177054
H	0.6463032	3.3228424	1.2081726

Cartesian coordinates of peptide backbones

ATSP-c0

H	1.26100000	0.29300000	5.48500000
C	0.90100000	0.72400000	6.41900000
H	1.74400000	0.66500000	7.10800000
H	0.08700000	0.09800000	6.78600000
C	0.49400000	2.18700000	6.31800000
O	-0.56700000	2.49800000	6.87200000
N	1.33800000	3.05300000	5.70200000
H	2.18200000	2.60300000	5.37900000
C	1.16100000	4.49900000	5.40300000
H	1.07900000	5.08700000	6.31700000
C	-0.19400000	4.72500000	4.58900000
O	-0.61000000	3.88200000	3.83100000
N	-0.73100000	5.94600000	4.73900000
H	-0.27800000	6.63600000	5.32200000
C	-1.95300000	6.40200000	4.13300000
H	-2.73000000	5.65000000	3.99800000
C	-1.78900000	6.78800000	2.65700000
O	-2.41800000	7.74600000	2.19800000
N	-0.99600000	6.01100000	1.94400000
H	-0.77600000	5.07300000	2.24700000
C	-0.71800000	6.30400000	0.56800000
H	-1.14200000	7.26000000	0.26100000
C	-1.31500000	5.29000000	-0.41500000
O	-0.73800000	4.14400000	-0.46600000
O	-2.14500000	2.66300000	-2.91300000
C	-2.28000000	3.90800000	-2.94300000
C	-3.17100000	4.76700000	-2.01100000
C	-4.04900000	3.94800000	-1.08200000
N	-2.40700000	5.64600000	-1.10000000
C	-5.26400000	3.06700000	-1.70300000
C	-6.21700000	2.41700000	-0.56300000
C	-5.50200000	1.54300000	0.49600000
C	-5.04600000	0.17800000	0.02900000
C	-6.16300000	-0.75300000	-0.34400000
C	-5.64700000	-1.98900000	-0.94000000
H	-3.40100000	3.30000000	-0.48000000
H	-4.67700000	4.48600000	-0.36100000
H	-2.72800000	6.60500000	-1.04700000
H	-4.86100000	2.22100000	-2.27300000
H	-5.85700000	3.68700000	-2.38600000
H	-6.68800000	3.31600000	-0.14500000
H	-7.10100000	1.96600000	-1.02800000
H	-4.65500000	2.10600000	0.90900000
H	-6.20100000	1.55700000	1.34200000
H	-4.59100000	-0.37100000	0.86300000
H	-4.33500000	0.19300000	-0.80700000
H	-6.87900000	-0.29900000	-1.04000000
H	-6.68000000	-1.05100000	0.57600000
H	-5.45400000	-1.93900000	-2.00900000
N	-1.54100000	4.54300000	-3.77400000
H	-1.66500000	5.54500000	-3.75300000
C	-0.57100000	4.11800000	-4.81700000
H	-1.11000000	3.40000000	-5.43400000
C	0.58000000	3.27200000	-4.31600000
O	1.20400000	2.55200000	-5.17700000
N	0.91600000	3.35400000	-3.02700000
H	0.29200000	3.95800000	-2.51100000
C	1.91100000	2.51600000	-2.49000000
H	2.53600000	2.31000000	-3.35900000
C	1.27100000	1.25900000	-1.91400000
O	1.62200000	0.17500000	-2.26300000
N	0.21600000	1.39200000	-1.02800000
H	-0.23200000	2.28800000	-0.90000000
C	-0.28000000	0.34200000	-0.17500000
H	0.55000000	-0.14800000	0.33400000
C	-1.04200000	-0.73800000	-0.98600000
O	-0.96300000	-1.94300000	-0.76500000
N	-1.76900000	-0.30500000	-2.10300000
H	-1.89200000	0.68100000	-2.28400000
C	-2.36200000	-1.18000000	-3.13600000
H	-2.99800000	-1.82000000	-2.52500000
C	-1.30400000	-2.09500000	-3.88400000
O	-1.69800000	-3.02600000	-4.58300000

N	0.01300000	-1.96000000	-3.61900000
H	0.34800000	-1.11900000	-3.17100000
C	0.97500000	-2.92100000	-4.13500000
H	0.60500000	-3.54400000	-4.94900000
C	1.34200000	-4.00200000	-2.97100000
O	1.29800000	-5.22400000	-3.34800000
N	1.77300000	-3.53800000	-1.81100000
C	2.18800000	-4.40600000	-0.62900000
C	1.00600000	-5.23800000	0.01000000
O	1.20400000	-6.32300000	0.56600000
H	3.00200000	-5.10000000	-0.87100000
H	1.84000000	-2.54000000	-1.65300000
C	-1.37800000	-6.83400000	-0.08100000
N	-0.24700000	-4.65800000	0.06200000
O	-1.61000000	-7.74500000	0.64700000
C	-1.49500000	-5.41900000	0.42300000
C	-2.63400000	-4.56800000	-0.22700000
C	-5.13400000	-4.43800000	-1.02100000
C	-5.50800000	-3.10900000	-0.31400000
C	-4.09000000	-5.24300000	-0.31500000
H	-0.31000000	-3.74700000	-0.37600000
H	-2.31000000	-4.48600000	-1.27100000
H	-2.71500000	-3.61300000	0.30600000
H	-4.68200000	-4.29300000	-2.01000000
H	-6.03900000	-5.05600000	-0.99100000
H	-5.77300000	-3.24400000	0.73200000
H	-4.46300000	-5.54900000	0.67000000
H	-4.02500000	-6.16100000	-0.91300000
N	-1.16600000	-7.01400000	-1.39900000
H	-0.98000000	-6.16700000	-1.91700000
C	-1.14500000	-8.26900000	-2.08000000
H	-2.11900000	-8.74900000	-1.97800000
C	-0.00100000	-9.26600000	-1.70600000
O	-0.07200000	-10.48400000	-1.93200000
N	0.92300000	-8.74600000	-1.00200000
H	0.91300000	-7.83200000	-0.57300000
C	2.20900000	-9.41500000	-0.64200000
H	2.39000000	-10.25300000	-1.31400000
C	2.27200000	-9.84500000	0.82800000
O	3.10400000	-10.69300000	1.11800000
N	1.46300000	-9.20300000	1.67500000
H	1.05200000	-8.37100000	1.27700000
C	1.43100000	-9.37900000	3.15700000
H	1.94100000	-10.31500000	3.38600000
C	0.02000000	-9.52300000	3.72500000
O	-0.12800000	-9.44900000	4.99000000
N	-0.98000000	-9.78400000	2.86100000
H	-0.79000000	-9.78300000	1.86900000
H	-1.91900000	-9.72600000	3.22900000
H	1.96973411	4.85407081	4.79903489
H	-2.27503370	7.26607750	4.67573784
H	0.34954566	6.32564416	0.49887834
H	-3.80552917	5.37112042	-2.62525667
H	-0.37405372	4.92589582	-5.49036209
H	2.45328800	3.11374927	-1.78749055
H	-3.01390130	-0.61651588	-3.77035821
H	-0.79738706	0.71878522	0.68246343
H	1.85324258	-2.45787529	-4.53388027
H	2.61133282	-3.80592922	0.14920588
H	-1.64578170	-5.37080493	1.48122593
H	-1.12785724	-8.02763000	-3.12227954
H	2.95811021	-8.71253768	-0.94246728
H	2.06950633	-8.66320446	3.63117973

ATSP-c1

H	-2.53200000	-1.03300000	5.32900000
C	-3.56400000	-0.68300000	5.32500000
H	-4.01900000	-0.74300000	6.31400000
H	-4.11700000	-1.24100000	4.57000000
C	-3.45000000	0.75300000	5.05900000
O	-4.44900000	1.48200000	5.33700000
N	-2.27200000	1.31500000	4.60200000
H	-1.58300000	0.61400000	4.37100000
C	-1.78300000	2.78700000	4.69900000
H	-2.40000000	3.20700000	5.49400000
C	-2.04200000	3.53900000	3.38200000
O	-2.00300000	3.00600000	2.26600000

N	-2.35900000	4.87900000	3.42800000
H	-2.47600000	5.30300000	4.33800000
C	-2.68700000	5.86100000	2.33600000
H	-3.17400000	5.33500000	1.51500000
C	-1.39900000	6.41400000	1.70400000
O	-1.57100000	7.21800000	0.77900000
N	-0.23300000	6.08900000	2.22700000
H	-0.18500000	5.59100000	3.10400000
C	1.12100000	6.60400000	1.77000000
H	0.98300000	7.60600000	1.36300000
C	1.57000000	5.73400000	0.57900000
O	2.16800000	4.67900000	0.85000000
O	3.35900000	4.37800000	-2.92400000
C	2.85100000	5.29300000	-2.30200000
C	1.33800000	5.40200000	-1.98900000
C	0.68900000	3.99100000	-1.93900000
N	1.05700000	6.05300000	-0.67200000
C	-0.81400000	3.88000000	-1.56800000
C	-1.35400000	2.49600000	-1.95700000
C	-2.75400000	2.19500000	-1.36000000
C	-3.84400000	3.25700000	-1.75200000
C	-4.14700000	3.32800000	-3.28100000
C	-4.91200000	2.13900000	-3.78800000
H	0.89800000	3.50500000	-2.89900000
H	1.33600000	3.40300000	-1.27700000
H	0.38400000	6.80400000	-0.76900000
H	-1.39300000	4.69800000	-2.01500000
H	-0.95900000	3.96900000	-0.48500000
H	-1.36000000	2.37300000	-3.04700000
H	-0.65500000	1.75000000	-1.55900000
H	-3.12900000	1.20200000	-1.63600000
H	-2.55600000	2.20000000	-0.28100000
H	-3.72600000	4.23100000	-1.26100000
H	-4.82300000	2.93300000	-1.37700000
H	-3.19000000	3.40000000	-3.81200000
H	-4.66400000	4.27600000	-3.47600000
H	-4.31100000	1.35600000	-4.24600000
N	3.64400000	6.24300000	-1.70200000
H	3.08300000	6.88300000	-1.15900000
C	5.10800000	6.37300000	-1.53800000
H	5.55100000	6.33800000	-2.53300000
C	5.67400000	5.18600000	-0.73300000
O	6.61500000	4.54300000	-1.23200000
N	5.09300000	4.87800000	0.41700000
H	4.34600000	5.51700000	0.64800000
C	5.62700000	3.85200000	1.35800000
H	6.63600000	3.62500000	1.01300000
C	4.94700000	2.52500000	1.26700000
O	5.64600000	1.49800000	1.43200000
N	3.64100000	2.42300000	0.90200000
H	3.23700000	3.29800000	0.60000000
C	2.82900000	1.21800000	0.93200000
H	3.34900000	0.51900000	1.58600000
C	2.57000000	0.55300000	-0.44200000
O	2.93900000	1.14200000	-1.48900000
N	1.97600000	-0.59800000	-0.51300000
H	1.70600000	-1.07000000	0.33900000
C	1.78200000	-1.39300000	-1.77100000
H	2.38000000	-1.01400000	-2.60000000
C	0.35900000	-1.35000000	-2.34100000
O	-0.63300000	-1.30900000	-1.62400000
N	0.25300000	-1.46300000	-3.71000000
H	1.06400000	-1.52900000	-4.30800000
C	-0.98600000	-1.32700000	-4.44700000
H	-1.48800000	-0.43900000	-4.06400000
C	-1.97000000	-2.46400000	-4.44400000
O	-2.45900000	-3.00000000	-5.48700000
N	-2.35700000	-3.00600000	-3.32100000
C	-2.95900000	-4.42200000	-3.20500000
C	-4.45700000	-4.47000000	-3.17300000
O	-5.07100000	-5.53400000	-2.95500000
H	-2.76800000	-4.96200000	-4.14000000
H	-1.87700000	-2.67100000	-2.49500000
C	-7.10800000	-4.35400000	-4.45600000
N	-5.07600000	-3.28500000	-3.47600000
O	-8.24300000	-4.83100000	-4.33600000
C	-6.56000000	-3.14700000	-3.55500000

C	-6.91200000	-1.80300000	-4.22800000
C	-6.87300000	0.71000000	-4.32900000
C	-6.21400000	1.94800000	-3.80000000
C	-6.55500000	-0.49400000	-3.45700000
H	-4.43200000	-2.50500000	-3.54100000
H	-7.96800000	-1.83500000	-4.52400000
H	-6.40900000	-1.74100000	-5.20100000
H	-6.46100000	0.51200000	-5.32600000
H	-7.95900000	0.84800000	-4.39200000
H	-6.94800000	2.62800000	-3.37400000
H	-5.48400000	-0.52200000	-3.21900000
H	-6.92700000	-0.45100000	-2.42600000
N	-6.39200000	-4.79200000	-5.55100000
H	-5.45600000	-4.42000000	-5.62800000
C	-6.92600000	-5.73600000	-6.51400000
H	-8.01100000	-5.67300000	-6.60200000
C	-6.56300000	-7.23200000	-6.14900000
O	-6.68600000	-8.00500000	-7.05000000
N	-6.06100000	-7.54200000	-4.94000000
H	-5.77200000	-6.85500000	-4.25700000
C	-5.84000000	-8.95100000	-4.52100000
H	-6.53500000	-9.51900000	-5.14100000
C	-6.24000000	-9.20100000	-3.05200000
O	-6.13000000	-10.33200000	-2.51700000
N	-6.86900000	-8.19500000	-2.39200000
H	-7.26700000	-7.39300000	-2.86000000
C	-7.29300000	-8.40700000	-1.03900000
H	-6.37100000	-8.56100000	-0.47900000
C	-8.32400000	-9.53800000	-0.72300000
O	-9.16900000	-9.95400000	-1.54700000
N	-8.30300000	-10.08400000	0.49100000
H	-7.66300000	-9.90500000	1.25200000
H	-9.06200000	-10.74100000	0.60300000
H	-7.62611395	-7.46659878	-0.65224344
H	-4.79443551	-9.13035880	-4.66073302
H	-6.54366678	-5.38439637	-7.44946576
H	-2.52845223	-5.00108675	-2.41494484
H	-7.04401455	-3.23712685	-2.60499628
H	-0.74991402	-1.04110398	-5.45070657
H	1.98838198	-2.41168970	-1.51687842
H	0.87749974	5.96609611	-2.77298666
H	5.39658940	7.28209154	-1.05305801
H	5.76946843	4.26168533	2.33614144
H	-3.36197103	6.58027683	2.75067451
H	-0.77213132	2.84296505	5.04528373
H	1.84813825	6.62912324	2.55456280
H	1.90936576	1.48167638	1.41121565

ATSP-c2

H	7.43200000	-5.62200000	-3.29400000
C	8.31300000	-5.50200000	-2.66300000
H	9.25700000	-5.72600000	-3.16100000
H	8.35200000	-6.29600000	-1.91700000
C	8.20100000	-4.21400000	-1.93900000
O	7.10300000	-3.67200000	-1.82200000
N	9.34400000	-3.66600000	-1.45000000
H	10.17300000	-4.24200000	-1.42200000
C	9.37500000	-2.43300000	-0.62100000
H	8.75900000	-2.70900000	0.23400000
C	8.79700000	-1.19800000	-1.31400000
O	8.16800000	-0.30800000	-0.69500000
N	8.92600000	-1.20500000	-2.62000000
H	9.49200000	-1.94300000	-3.01500000
C	8.51200000	-0.07900000	-3.51400000
H	8.82800000	0.84300000	-3.02600000
C	7.03300000	0.14300000	-3.79100000
O	6.56600000	0.94700000	-4.57100000
N	6.25700000	-0.78200000	-3.18200000
H	6.69900000	-1.24100000	-2.39800000
C	4.80200000	-1.11800000	-3.32800000
H	4.27300000	-0.35500000	-3.89900000
C	4.06500000	-1.19800000	-1.94500000
O	3.29200000	-0.29700000	-1.63900000
O	2.48800000	-0.42400000	1.37400000
C	3.60400000	-0.67800000	1.02000000
C	4.10200000	-2.12000000	0.36500000

C	2.90100000	-3.21800000	0.34900000
N	4.61900000	-1.94100000	-1.01200000
C	2.38700000	-3.68400000	1.72100000
C	1.16600000	-4.60700000	1.51400000
C	0.71100000	-5.13300000	2.86500000
C	-0.35500000	-6.28800000	2.70700000
C	-1.74900000	-5.93700000	2.04400000
C	-2.64500000	-5.08300000	2.99400000
H	2.08100000	-2.79100000	-0.24000000
H	3.19900000	-4.07800000	-0.26300000
H	5.46600000	-2.43500000	-1.26500000
H	2.07500000	-2.77700000	2.25400000
H	3.17500000	-4.14300000	2.33000000
H	0.41700000	-4.10500000	0.88900000
H	1.46700000	-5.46600000	0.90100000
H	1.48900000	-5.42900000	3.57800000
H	0.25300000	-4.29700000	3.40900000
H	-0.52300000	-6.70100000	3.70900000
H	0.02500000	-7.12000000	2.10300000
H	-2.20900000	-6.91600000	1.86300000
H	-1.64100000	-5.43000000	1.07700000
H	-2.76100000	-5.45300000	4.01100000
N	4.56400000	0.20100000	1.30500000
H	5.49100000	0.00300000	0.95700000
C	4.30500000	1.35300000	2.12800000
H	3.75100000	0.98000000	2.99000000
C	3.43500000	2.45000000	1.37900000
O	3.13300000	3.50500000	1.91500000
N	3.15500000	2.23500000	0.11900000
H	3.68200000	1.46100000	-0.25800000
C	2.29200000	3.02600000	-0.79900000
H	2.19700000	4.01400000	-0.34700000
C	0.85300000	2.41300000	-0.93400000
O	-0.14600000	3.07900000	-0.62300000
N	0.84500000	1.15900000	-1.23700000
H	1.70100000	0.63300000	-1.13000000
C	-0.36700000	0.29600000	-1.23900000
H	-1.17500000	0.82100000	-1.74800000
C	-1.09700000	0.14900000	0.14200000
O	-2.32500000	0.15500000	0.11800000
N	-0.45200000	-0.07700000	1.32500000
H	0.55300000	-0.12300000	1.23500000
C	-1.01400000	-0.08100000	2.63900000
H	-1.74500000	-0.89000000	2.65000000
C	-1.70400000	1.20400000	3.03900000
O	-2.46300000	1.16700000	3.98800000
N	-1.55000000	2.36100000	2.39800000
H	-0.99700000	2.36900000	1.55300000
C	-2.25900000	3.58000000	2.67100000
H	-2.50800000	3.70600000	3.72400000
C	-3.63900000	3.75300000	2.02400000
O	-4.64400000	3.84000000	2.61300000
N	-3.61400000	3.39400000	0.72400000
C	-4.78000000	3.19800000	-0.14600000
C	-5.71400000	2.13200000	0.46800000
O	-6.92400000	2.25400000	0.46000000
H	-5.39200000	4.10800000	-0.14900000
H	-2.75400000	3.12900000	0.26000000
C	-7.08300000	0.14600000	2.40800000
N	-5.16400000	0.91500000	0.99000000
O	-8.23100000	-0.16700000	2.39900000
C	-5.93000000	-0.27000000	1.42000000
C	-4.92500000	-1.23500000	2.15600000
C	-4.27400000	-3.33000000	3.64400000
C	-3.32000000	-3.91900000	2.68200000
C	-5.41800000	-2.49200000	2.83500000
H	-4.15200000	0.86400000	0.99000000
H	-4.31300000	-0.68500000	2.88200000
H	-4.16400000	-1.58500000	1.44800000
H	-3.78700000	-2.56900000	4.26500000
H	-4.64800000	-4.14300000	4.27700000
H	-3.18200000	-3.43300000	1.71800000
H	-5.88700000	-3.22900000	2.17200000
H	-6.25600000	-2.35100000	3.52800000
N	-6.63600000	1.04800000	3.36500000
H	-5.63900000	1.21000000	3.36100000
C	-7.32800000	1.70300000	4.43900000

H	-8.06000000	1.05600000	4.92200000
C	-8.11700000	2.96300000	4.06200000
O	-8.57700000	3.66100000	4.94800000
N	-8.32100000	3.24400000	2.75100000
H	-7.85200000	2.66400000	2.07100000
C	-9.11700000	4.34300000	2.18300000
H	-9.69900000	4.86800000	2.94000000
C	-10.24100000	3.92900000	1.25800000
O	-11.21100000	4.62200000	1.08000000
N	-10.08700000	2.71400000	0.70900000
H	-9.32500000	2.11400000	0.99200000
C	-11.05600000	2.07600000	-0.13200000
H	-11.84100000	2.80300000	-0.34200000
C	-11.91300000	0.86400000	0.35200000
O	-12.82600000	0.37200000	-0.24200000
N	-11.45000000	0.35400000	1.53000000
H	-10.69200000	0.75800000	2.06100000
H	-11.99300000	-0.41100000	1.90400000
H	10.34399927	-2.19267141	-0.23605508
H	9.02539877	-0.08039700	-4.45278632
H	4.85131893	-2.58992392	0.96715667
H	2.78733225	3.12057580	-1.74271679
H	5.19039622	1.86999470	2.43408825
H	-0.19148366	-0.62957444	-1.74635192
H	-0.20548409	-0.20287970	3.32917923
H	-1.64978665	4.42972858	2.44357873
H	-4.42928560	2.88809852	-1.10821644
H	-10.54983789	1.77849514	-1.02653383
H	-8.43423098	5.00819279	1.69694958
H	-6.61570915	2.07491041	5.14555813
H	-6.29040500	-0.78631460	0.55488297
H	4.65227790	-1.98595420	-3.93556794

p53-c0

C	9.97492637	-4.95637350	2.91493578
H	9.72881535	-4.15965078	3.61736828
C	8.74047825	-5.11217982	2.03808430
O	8.51582626	-6.19327859	1.49884027
N	7.89576943	-4.10150109	1.82397724
H	7.90245575	-3.26101250	2.38521461
C	6.85726411	-4.19395789	0.81873861
H	6.53261826	-5.22449272	0.67461296
C	7.26906238	-3.64437732	-0.53964857
O	8.07116343	-2.71328223	-0.57877774
N	6.68872428	-4.25710402	-1.57364613
H	5.83764391	-4.76536604	-1.38096484
C	7.05452594	-3.99742130	-2.95154138
H	8.13176518	-4.09372113	-3.08647151
C	6.56271453	-2.70133831	-3.58097109
O	6.82157386	-2.32641803	-4.72158665
N	5.75179104	-1.95339534	-2.82832398
H	5.44176424	-2.24668419	-1.91270443
C	5.25834046	-0.65011590	-3.22353471
H	6.05896351	-0.19607266	-3.80745046
C	5.00206113	0.23851000	-2.01437994
O	4.53555956	-0.21627271	-0.97209390
N	5.07382397	1.55556398	-2.21665001
H	5.38888334	1.86063932	-3.12654659
C	4.64733447	2.50224931	-1.20629567
H	5.03161156	2.15847966	-0.24578918
C	3.13264787	2.49389353	-1.05616568
O	2.62563770	3.07842335	-0.10246668
N	2.41003375	1.96968734	-2.04898489
H	3.01907582	1.66651166	-2.79538855
C	0.97859243	1.80383873	-2.20248173
H	0.63282291	2.83798361	-2.19161989
C	0.13886413	1.20369039	-1.08390241
O	-0.97223570	1.54150003	-0.68060594
N	0.76368586	0.20572768	-0.45572529
H	1.68161238	-0.07717102	-0.76799502
C	0.34928395	-0.35307788	0.81526554
H	-0.73467429	-0.38504944	0.92864782
C	0.83765055	0.35014881	2.07417642
O	0.04667504	0.59023124	2.98312383
N	2.06959478	0.86448214	2.03381677
H	2.49190873	0.83789646	1.11681825
C	2.67740181	1.51570762	3.17632378

H	2.50155140	0.97496292	4.10651408
C	2.19404942	2.91337350	3.53608743
O	2.38541222	3.47401791	4.61278951
N	1.59035415	3.59702608	2.56128826
H	1.63247623	3.21950574	1.62500131
C	0.92149714	4.88211630	2.58406410
H	1.37021649	5.62306698	3.24484035
C	-0.56227494	4.70564110	2.87082922
O	-1.37316048	5.62867102	2.81954931
N	-1.06519234	3.46948712	2.83673100
H	-0.43775556	2.68312198	2.92390380
C	-2.42899908	3.03230382	2.61835688
H	-2.50265615	2.06112294	2.12831249
C	-3.13834149	3.85995993	1.55590686
O	-4.32250906	4.14232962	1.72805722
N	-2.49579343	3.98264266	0.39328689
H	-1.56709674	3.58875850	0.42313447
C	-3.12730098	4.31276420	-0.86897587
H	-3.67776763	5.23204991	-0.67343196
C	-4.04509644	3.22796289	-1.41382809
O	-5.12091341	3.56881003	-1.89946918
N	-3.66356681	1.95321108	-1.52953996
H	-2.80376205	1.68103655	-1.07435875
C	-4.33774829	0.91092329	-2.27692327
H	-4.54210416	1.36254614	-3.24733176
C	-5.64146430	0.62884242	-1.54378465
O	-5.56494818	0.38028118	-0.34203226
N	-6.80226974	0.64255274	-2.20302438
C	-6.93955435	0.92771476	-3.61665547
H	-6.21561855	0.33787956	-4.18019919
H	-6.90643859	2.01156963	-3.73416358
C	-8.33127601	0.40731364	-3.96427568
H	-8.27013493	-0.58921873	-4.40182475
H	-8.79555523	1.05768996	-4.70639941
C	-9.07560826	0.45840903	-2.63286249
H	-9.88639084	-0.26703203	-2.57944844
H	-9.40134660	1.48254624	-2.45125932
C	-8.00462617	0.09863263	-1.60616654
H	-8.32618766	0.44216869	-0.62255415
C	-7.92728681	-1.41326825	-1.44754532
O	-7.83724677	-2.08532161	-2.47218492
N	-8.04449655	-1.94214541	-0.22694421
H	-8.10035163	-1.31900210	0.56607456
C	-7.55394210	-3.24974844	0.16014748
H	-6.64865461	-3.56935692	-0.35493904
C	-8.62925943	-4.28033328	-0.15637724
O	-8.47189217	-5.28664826	-0.84326508
N	-9.76651007	-3.94871449	0.45932172
H	-9.94759474	-2.96297433	0.58144394
C	-10.96247150	-4.76754191	0.44478513
H	-10.64755749	-5.77926491	0.70161754
H	-7.31511249	-3.30129703	1.20187833
H	-3.72993904	0.03041984	-2.26344734
H	-2.33361657	4.57176609	-1.53821655
H	0.70113082	-1.36036863	0.89569640
H	0.75310098	1.46409914	-3.19173934
H	0.96036486	5.31191359	1.60495043
H	-2.91617479	2.93982578	3.56651701
H	3.71969705	1.58348603	2.94410247
H	6.03044402	-3.63713338	1.20760236
H	4.43702837	-0.79949291	-3.89288735
H	10.77654336	-4.56834806	2.32185361
H	10.33054991	-5.86112496	3.36198913
H	-11.62084315	-4.48212524	1.23850051
H	6.66190156	-4.80832473	-3.52876033
H	-11.43572087	-4.83859963	-0.51223411
H	5.18169694	3.40614883	-1.41201981

pP53-c1

C	-9.99517608	6.44080834	2.90503139
H	-10.42158123	5.62746925	3.49294585
C	-8.92771222	5.83196181	2.00616484
O	-8.18003573	6.49684080	1.29214590
N	-8.85776584	4.49893939	1.96988817
H	-9.66407755	4.03967246	2.36657507
C	-7.83158754	3.69763129	1.33416046
H	-6.93295016	4.24407439	1.62265481

C	-7.97219829	3.51384364	-0.17080667
O	-9.07364422	3.22863768	-0.63562988
N	-6.93480435	3.77210737	-0.96942981
H	-6.08285438	3.99715155	-0.47614032
C	-6.97817354	4.06619005	-2.38822818
H	-8.01435366	3.94327839	-2.70443799
C	-6.13917402	3.05845741	-3.16117842
O	-6.11653736	3.01396621	-4.38915494
N	-5.50087582	2.11581269	-2.46257512
H	-5.49496872	2.16378312	-1.45395026
C	-4.67079415	1.02862531	-2.93998615
H	-5.05971244	0.73118631	-3.91376989
C	-4.73888152	-0.20826388	-2.05497284
O	-4.79462918	-0.03881284	-0.83877353
N	-4.67608640	-1.40895054	-2.63412532
H	-4.83743527	-1.47486296	-3.62994570
C	-4.50197881	-2.59947054	-1.82680693
H	-5.02652207	-2.47598650	-0.87966480
C	-3.06264372	-2.88444349	-1.42212525
O	-2.76061515	-3.62342106	-0.48752100
N	-2.07303754	-2.33393714	-2.12990461
H	-2.38672780	-1.74872333	-2.89062754
C	-0.64206299	-2.46721164	-1.95091010
H	-0.48270540	-3.53791830	-2.08038055
C	-0.22594442	-2.02580879	-0.55562833
O	0.87423379	-2.36783422	-0.12788924
N	-1.05629771	-1.33593212	0.23145860
H	-1.94801666	-1.21961342	-0.22723566
C	-0.85501402	-1.08933782	1.64551851
H	0.04703689	-0.51210845	1.84927471
C	-0.72931678	-2.34923384	2.48940744
O	0.10707972	-2.39503622	3.38840035
N	-1.49936078	-3.38198678	2.13807591
H	-1.78529684	-3.33231211	1.17035636
C	-1.37198640	-4.68053700	2.76869260
H	-1.31956612	-4.49017044	3.83987040
C	-0.10174471	-5.38016859	2.30586649
O	0.51393586	-6.16521626	3.02460970
N	0.29024304	-5.15766290	1.04938605
H	-0.37901023	-4.70548336	0.44312302
C	1.54592991	-5.63622222	0.50768526
H	1.55103842	-6.71769806	0.64437502
C	2.74603124	-4.99864851	1.19456728
O	3.81844913	-5.59732256	1.14013845
N	2.54253465	-3.81442963	1.77634820
H	1.65802865	-3.34846353	1.63258838
C	3.61671805	-2.88120301	2.05116339
H	3.07700728	-1.93605020	2.12210431
C	4.60885732	-2.55369391	0.94358053
O	5.76520383	-2.22542019	1.19680245
N	4.29162457	-2.60615013	-0.35146580
H	3.31191544	-2.70504174	-0.57404300
C	5.17293293	-2.37314662	-1.47821122
H	6.16614545	-2.23548856	-1.05081989
C	4.88988956	-1.04134841	-2.15841362
O	4.94694580	-0.94208844	-3.38253055
N	4.65969918	-0.03162505	-1.31579604
H	4.68163649	-0.31069652	-0.34528482
C	4.36199322	1.35818259	-1.59757286
H	3.80331998	1.36434018	-2.53339094
C	5.61113604	2.19589394	-1.83099784
O	6.52221977	2.05197040	-1.01845758
N	5.68946480	3.10266056	-2.80747270
C	4.74838785	3.50196515	-3.83479411
H	3.71820407	3.38189573	-3.49833477
H	4.90867862	2.82101630	-4.67010526
C	4.98508445	4.94725477	-4.26308953
H	4.66989864	5.52988405	-3.39850742
H	4.49477429	5.35912374	-5.14566478
C	6.49648493	4.75150042	-4.33818348
H	7.08501623	5.66862258	-4.32480414
H	6.71894141	4.15050119	-5.21976003
C	6.83567781	3.94254687	-3.08905070
H	7.64814639	3.27021978	-3.36343773
C	7.41576392	4.81423321	-1.98432840
O	8.57698714	5.20980293	-2.05853354
N	6.67701655	5.16644294	-0.92960287

H	5.73340477	4.81311460	-0.85963102
C	7.00018795	5.98726385	0.22042434
H	7.62684822	6.81653889	-0.10930427
C	7.82334380	5.14572331	1.18523173
O	8.47331735	5.67632560	2.08312696
N	8.00635616	3.83997375	0.97686709
H	7.73880604	3.43135818	0.09281370
C	8.44044793	2.84122554	1.93188051
H	8.43447870	3.31253578	2.91594159
H	-3.65694241	1.35013899	-3.05674917
H	-6.72685921	5.09415994	-2.54640217
H	-7.73767563	2.73310795	1.78778505
H	-10.73612014	6.82587361	2.23598233
H	-9.64063031	7.22828237	3.53676072
H	-0.10309558	-2.01481636	-2.75698246
H	-2.24728125	-5.24901877	2.53292279
H	1.49761983	-5.45460179	-0.54568085
H	4.10084051	-3.09093325	2.98204398
H	9.40765472	2.45846589	1.68106969
H	3.72569355	1.74926753	-0.83136447
H	6.16129333	6.38790701	0.75017983
H	-1.73583082	-0.55556317	1.93559848
H	-4.93318697	-3.42113956	-2.35955410
H	5.17640422	-3.23830052	-2.10781163
H	7.70909335	2.06161931	1.97915206

p53-c2

C	-12.45303358	3.22102342	0.78817779
H	-12.13443872	4.17212055	0.35933546
C	-12.27360180	2.11044783	-0.23583757
O	-13.23908882	1.48517173	-0.66812089
N	-11.04561308	1.73851393	-0.60383622
H	-10.32507274	2.40350985	-0.35920543
C	-10.46528540	0.57169032	-1.23732798
H	-11.10535815	-0.28050681	-1.01174848
C	-9.14991443	0.15701322	-0.59477653
O	-8.51362298	0.95507724	0.09056736
N	-8.65205765	-1.07173640	-0.75519561
H	-9.24527149	-1.73162700	-1.23885550
C	-7.58522563	-1.74940180	-0.04754008
H	-7.06092300	-0.97108762	0.50640572
C	-6.59459037	-2.37992140	-1.01630893
O	-6.95527848	-3.23669618	-1.81995703
N	-5.28754423	-2.21124658	-0.80834384
H	-5.00575705	-1.73365691	0.03669653
C	-4.31036176	-2.88894189	-1.63703244
H	-4.77182602	-3.68575621	-2.22018581
C	-3.13038432	-3.38115716	-0.81183811
O	-2.87466350	-3.07709983	0.35114737
N	-2.26573214	-4.19386513	-1.42419472
H	-2.51858975	-4.48910409	-2.35632300
C	-1.08763116	-4.82663181	-0.86666022
H	-1.31365529	-5.04770225	0.17604353
C	0.13366425	-3.93110122	-1.01960732
O	1.10941704	-4.11066542	-0.29484017
N	0.10540627	-2.78716855	-1.70807940
H	-0.60637883	-2.67420721	-2.41601996
C	1.22946115	-1.87460266	-1.76576327
H	2.13004362	-2.48506765	-1.70266903
C	1.36145855	-0.97326509	-0.54711911
O	2.44199624	-0.47592395	-0.23841971
N	0.24197099	-0.72546053	0.13786757
H	-0.59077358	-1.22883631	-0.13239795
C	0.21688779	0.02562542	1.37654026
H	0.92223568	0.85391053	1.30517286
C	0.64566435	-0.77094088	2.60075066
O	1.18683302	-0.20753953	3.54890077
N	0.32208574	-2.06150125	2.49551723
H	-0.27457295	-2.23408519	1.69927710
C	0.69393863	-3.07500922	3.46097480
H	0.29194129	-2.77416126	4.42953911
C	2.19030389	-3.34696563	3.40011388
O	2.74883896	-3.34570995	4.49483724
N	2.74554141	-3.46131238	2.19110050
H	2.20020541	-3.29691515	1.35750146
C	4.12353449	-3.84002751	1.94921578
H	4.50277327	-4.31187618	2.85562516

C	4.98574036	-2.60300197	1.74305274
O	6.18356877	-2.72310546	1.49789464
N	4.46245015	-1.39719298	1.97572048
H	3.45868458	-1.44117435	2.08324941
C	5.14508851	-0.14906323	1.70124774
H	4.32420850	0.50882515	1.41687641
C	6.00243917	-0.10329633	0.44492392
O	7.07391504	0.49799601	0.44128849
N	5.49165829	-0.71182421	-0.62879761
H	4.60640622	-1.16394323	-0.44828077
C	5.83815271	-0.39867836	-2.00043152
H	6.91782712	-0.28449938	-2.09976652
C	5.18172503	0.86694025	-2.53176530
O	5.83739318	1.66871520	-3.19343701
N	3.94634864	1.16038725	-2.11844305
H	3.57527898	0.58830245	-1.37308042
C	3.37929563	2.46737625	-2.38255842
H	3.27652245	2.59526928	-3.43990465
C	4.25353963	3.58478002	-1.83092214
O	4.54808206	3.46697505	-0.64403884
N	4.72885288	4.56373167	-2.60424306
C	4.66841998	4.64537554	-4.05016714
H	3.78441686	5.24560942	-4.26414363
H	4.58041067	3.68062141	-4.55162202
C	5.96892289	5.29736716	-4.50903573
H	5.93073354	5.68723496	-5.52611334
H	6.79091571	4.58279560	-4.45513301
C	6.08484567	6.30653363	-3.36985563
H	5.40386038	7.06628717	-3.75063217
H	7.05182581	6.71633934	-3.07753408
C	5.63185560	5.58100696	-2.10658116
H	6.48865464	5.16720286	-1.57455136
C	5.01833993	6.56680946	-1.12203539
O	5.68677738	7.05090779	-0.21207805
N	3.72422616	6.84191035	-1.29957216
H	3.23749976	6.31314120	-2.01044255
C	2.90948701	7.76254086	-0.53219160
H	3.52248548	8.44201043	0.06078999
C	1.95036979	7.05564234	0.41449262
O	1.15431680	6.20808158	0.01751713
N	2.11796003	7.36847092	1.70161541
H	2.83677187	8.07502881	1.76411817
C	1.61023349	6.68235330	2.87208443
H	0.97164545	5.89350190	2.47520175
H	-11.95532715	2.99899488	1.70898811
H	-13.49908666	3.39448563	0.93164846
H	-10.39516317	0.75820671	-2.28861030
H	-8.01387291	-2.37608837	0.70639987
H	-4.01101219	-2.14415913	-2.34455522
H	-0.95008224	-5.78423778	-1.32379380
H	4.09256299	-4.51183323	1.11697913
H	-0.78233485	0.39553390	1.47463123
H	0.19642517	-3.95018337	3.09841370
H	1.14172361	-1.39441531	-2.71793040
H	5.56312724	0.27857713	2.58852862
H	5.50063145	-1.20782882	-2.61382776
H	2.45015728	6.24358940	3.36898911
H	0.97865440	7.30832070	3.46720851
H	2.42478685	8.44044483	-1.20331899
H	2.41205601	2.52558684	-1.92873130

p53-c3

C	11.35014136	-4.15133372	3.64046433
H	11.35437589	-3.15386023	4.08110920
C	10.28485898	-4.11387426	2.55468091
O	9.41492312	-4.97782505	2.46664777
N	10.23159337	-3.02434204	1.78509103
H	10.84873644	-2.24710049	1.97265471
C	9.29743693	-2.83788432	0.69378375
H	8.92747557	-3.82010076	0.39832884
C	9.94511184	-2.16397724	-0.50792327
O	10.73463181	-1.24062779	-0.32580994
N	9.54636583	-2.54249259	-1.72393107
H	8.90340573	-3.31662614	-1.81684896
C	10.07947888	-2.01444535	-2.96381529
H	11.08535122	-1.61084283	-2.84976688
C	9.30802471	-0.79695953	-3.45067881

O	9.06665665	-0.50100109	-4.61912233
N	8.64192872	-0.13555749	-2.50146823
H	8.75579984	-0.64978065	-1.63901008
C	7.78957637	1.02801993	-2.63044996
H	8.24391806	1.72632889	-3.33289667
C	7.62627396	1.81329400	-1.33673775
O	7.56920362	1.25530881	-0.24297693
N	7.64683197	3.14813239	-1.34827482
H	7.59098599	3.66173713	-2.21590005
C	7.33916144	4.01569058	-0.22929719
H	7.77344112	3.69185877	0.71677007
C	5.84620245	4.12204393	0.04765914
O	5.44515827	4.70720384	1.05121764
N	4.98163279	3.68172347	-0.86881149
H	5.21643185	3.34812039	-1.79262832
C	3.54067745	3.75162544	-0.73544458
H	3.37793788	4.67790389	-0.18519624
C	2.91688422	2.57898253	0.00820316
O	3.50918850	1.50437459	0.07441212
N	1.89855923	2.87059574	0.82069143
H	1.63847318	3.82848191	1.00359710
C	1.28920133	1.90677040	1.71519293
H	1.58248092	0.89719421	1.42797843
C	-0.22366038	1.98058946	1.57010492
O	-0.93063068	1.09628969	2.04925534
N	-0.78949648	2.94305419	0.83778982
H	-0.17112461	3.61915526	0.41298665
C	-2.20153770	3.17064264	0.60445662
H	-2.88442121	3.12037842	1.45187139
C	-2.82555985	2.12856598	-0.31357275
O	-4.02046803	1.86627827	-0.20510242
N	-2.00176135	1.42657002	-1.09560660
H	-1.02756418	1.69572668	-1.07635271
C	-2.51179223	0.43155406	-2.01668262
H	-3.31240508	0.91480594	-2.57686996
C	-3.12279539	-0.77670797	-1.32138459
O	-3.85895846	-1.57345820	-1.89932075
N	-2.78435560	-0.89695325	-0.03625353
H	-1.99701835	-0.30826791	0.19662579
C	-3.25400225	-1.92508700	0.87054430
H	-3.27473834	-2.89086961	0.36601368
C	-4.68039570	-1.70453733	1.35397155
O	-5.44848087	-2.60812843	1.67379267
N	-4.94604207	-0.39646381	1.38104422
H	-4.14305017	0.16476587	1.13615775
C	-6.10461684	0.19061389	2.02269410
H	-6.40847783	-0.64463780	2.65321555
C	-7.20854890	0.46302510	1.01084901
O	-8.33613815	0.28144892	1.46366775
N	-6.87727966	0.82870152	-0.22992643
H	-5.87656590	0.87234840	-0.35541790
C	-7.77562725	1.44313433	-1.18708796
H	-8.82170902	1.43644125	-0.88090292
C	-7.65182317	0.75794338	-2.54036740
O	-6.60641949	0.19320455	-2.85583436
N	-8.69326373	0.79127396	-3.37359482
C	-8.52033443	0.44214740	-4.76912892
H	-8.56569624	-0.64552694	-4.83134628
H	-7.55317500	0.58918223	-5.24857811
C	-9.64471760	1.12481023	-5.54416522
H	-10.15646200	0.55501668	-6.31920018
H	-9.29554841	2.04592824	-6.00903902
C	-10.68750608	1.40139067	-4.46386625
H	-11.43449867	0.60963073	-4.50929152
H	-11.06458514	2.41424810	-4.60794042
C	-10.02024278	1.30171625	-3.09468180
H	-10.05264034	2.28269880	-2.62082336
C	-10.87582556	0.49469574	-2.12900052
O	-11.92151054	0.89928601	-1.62702253
N	-10.45172910	-0.71336133	-1.74980198
H	-9.72958287	-1.08154114	-2.35355905
C	-10.94345531	-1.52527000	-0.65564296
H	-11.31443465	-0.94407615	0.18921337
C	-9.76452989	-2.33051646	-0.12859337
O	-8.94362887	-2.85878345	-0.87435254
N	-9.69099097	-2.57481599	1.18165890
H	-10.34519831	-2.08832147	1.77767402

C	-8.70519684	-3.32549320	1.93413146
H	-7.75075201	-3.14930085	1.43788411
H	-8.57501816	-2.96098260	2.93167120
H	-8.88574210	-4.37768245	1.86201210
H	-11.74312319	-2.13302992	-1.02450495
H	-2.53834148	-2.07664127	1.65141622
H	-5.79128090	1.02179239	2.61923654
H	-7.52264496	2.47801537	-1.28669311
H	-1.71991900	0.09794032	-2.65428646
H	1.72950294	2.08705994	2.67359273
H	-2.33177894	4.16956708	0.24378061
H	3.06317513	3.78550545	-1.69238955
H	7.79194803	4.95513124	-0.46874562
H	6.83579722	0.77055085	-3.04144267
H	10.08786536	-2.78106259	-3.71022515
H	8.46749846	-2.30730280	1.11161790
H	12.33939958	-4.27550293	3.25206998
H	11.10561459	-4.79185614	4.46194938

p53-c4

C	6.47845311	-3.54991020	4.60723249
H	5.66351093	-2.87092356	4.86009730
C	6.39213848	-3.95254883	3.14250411
O	6.51325905	-5.13062664	2.81162844
N	6.23003868	-3.01268940	2.20791427
H	6.08023410	-2.05327186	2.48487406
C	6.46591198	-3.16848835	0.78698260
H	7.05419573	-4.06396765	0.58849528
C	7.17171044	-1.97419388	0.16075056
O	7.13604040	-0.83572957	0.62204858
N	7.80712246	-2.07310513	-1.00885338
H	7.90913433	-3.00865152	-1.37659879
C	8.39881591	-0.97752125	-1.75000394
H	8.63590436	-0.19977242	-1.02347178
C	7.41938391	-0.21790313	-2.63273583
O	7.70580285	0.17571544	-3.76109342
N	6.24779554	0.13300746	-2.09635095
H	6.08606146	-0.13227830	-1.13541615
C	5.30422594	1.07532294	-2.66393116
H	5.82679810	1.75388897	-3.33814599
C	4.63993007	1.89470453	-1.56697213
O	4.38998951	1.35990025	-0.48822225
N	4.29836283	3.16524993	-1.79249475
H	4.63125782	3.63567802	-2.62148419
C	3.56950851	4.00960911	-0.86761516
H	3.94089054	3.82852042	0.14038456
C	2.07533896	3.72875320	-0.79407860
O	1.45164379	4.02236867	0.22369323
N	1.55952240	3.09189167	-1.84896340
H	2.10377678	2.84285610	-2.66206769
C	0.20767025	2.57064371	-1.83960185
H	-0.46704978	3.40768438	-1.65970750
C	-0.22508960	1.54977647	-0.79812055
O	-1.40925526	1.36324725	-0.52600887
N	0.74115965	0.88033736	-0.16550857
H	1.69321690	1.13517322	-0.39056821
C	0.61733396	-0.02480159	0.95933141
H	-0.29857412	-0.60794764	0.85573972
C	0.38206927	0.84544015	2.18579917
O	-0.68221602	0.83387266	2.79940918
N	1.30945824	1.79162336	2.34619954
H	2.09092563	1.73796115	1.70815311
C	1.35491551	2.81323139	3.37268868
H	1.08403297	2.24436551	4.26215506
C	0.30216042	3.91096669	3.32869729
O	0.42137993	4.86112418	4.09894639
N	-0.60726192	3.87080139	2.35146035
H	-0.50389403	3.04443676	1.78010869
C	-1.72905642	4.72043312	2.00489963
H	-1.88075686	5.43523377	2.81297151
C	-3.01249963	3.90359145	2.00829538
O	-4.11908624	4.41102182	1.83789076
N	-2.94491566	2.58114928	2.18276990
H	-2.06517451	2.11805905	2.36073089
C	-4.10211894	1.71487109	2.08426121
H	-3.60705528	0.74522053	2.03335995
C	-4.91033377	1.90656717	0.80961823

O	-6.13626879	1.85518483	0.87824366
N	-4.31512277	2.18075650	-0.35399740
H	-3.31102694	2.22764788	-0.25839974
C	-4.91806684	2.35220221	-1.66044989
H	-5.86169379	2.88870710	-1.57445085
C	-5.12374392	0.96285300	-2.24617596
O	-5.99464169	0.82813599	-3.10317630
N	-4.34749020	-0.06950883	-1.90769633
H	-3.77063887	0.04708379	-1.08622335
C	-4.47848572	-1.36909386	-2.53563681
H	-4.67436818	-1.08172964	-3.56838365
C	-5.53338593	-2.25817586	-1.89290118
O	-5.74003356	-2.05406252	-0.69794648
N	-6.09490988	-3.24305298	-2.59688283
C	-5.97381920	-3.52103889	-4.01319889
H	-5.02592663	-3.99127113	-4.27268208
H	-6.05078232	-2.56582449	-4.53237448
C	-7.13504692	-4.43872316	-4.38493135
H	-6.78358475	-5.46856071	-4.45338636
H	-7.59208747	-4.07306688	-5.30439617
C	-8.10050425	-4.20760291	-3.22491329
H	-8.85489568	-4.99016418	-3.15098330
H	-8.62814879	-3.26994744	-3.40152206
C	-7.19220452	-3.99324013	-2.01827934
H	-7.71883114	-3.39823179	-1.27196663
C	-6.92349128	-5.27495014	-1.24256613
O	-7.80237071	-5.82757848	-0.58393565
N	-5.67066118	-5.73426109	-1.28561727
H	-5.03595617	-5.29263658	-1.93497351
C	-5.12340494	-6.75953105	-0.42009694
H	-5.87375861	-7.54654992	-0.49721935
C	-5.08922879	-6.35106480	1.04625010
O	-5.02556676	-7.24006776	1.89202199
N	-5.12164765	-5.04504785	1.32164074
H	-5.32452459	-4.48761156	0.50477852
C	-5.17299608	-4.40470233	2.62092026
H	-4.73605473	-5.10514649	3.33223340
H	9.25495100	-1.36753875	-2.25972844
H	4.52304430	0.58739387	-3.20852319
H	3.80479070	5.03558270	-1.05976219
H	2.36570057	3.15887199	3.43389444
H	-0.06044800	2.24769484	-2.82383588
H	1.44790149	-0.69928546	0.97069299
H	-1.56392548	5.24648085	1.08789061
H	-4.67832899	1.67124190	2.98480464
H	-4.26050122	2.94597809	-2.26039794
H	-3.53311199	-1.87008327	-2.52229055
H	-4.72689298	-3.43342008	2.57089207
H	-6.19806723	-4.35008984	2.92282526
H	-4.14048046	-7.06709434	-0.71020776
H	7.32394588	-2.91742903	4.78046510
H	6.37446796	-4.39615268	5.25373133
H	5.47911233	-3.20478379	0.37490361

pDIQ-c0

C	-10.16284859	-1.53032868	1.20511692
H	-9.51835907	-2.39936278	1.34049480
C	-9.33104054	-0.44091381	1.86555005
O	-9.75121764	0.28571499	2.76372373
N	-8.07270906	-0.24435098	1.46502260
H	-7.78284116	-0.88574447	0.74130364
C	-7.20965615	0.89707708	1.69655433
H	-7.89056434	1.74596972	1.62367446
C	-6.19576413	0.95759003	0.56402903
O	-5.76305912	-0.06287924	0.03340107
N	-5.65171198	2.15253484	0.31817461
H	-6.05759902	2.89210624	0.87272307
C	-4.51216498	2.30301157	-0.56344362
H	-4.57458641	1.73181398	-1.49035411
C	-3.16745594	1.95958090	0.06232574
O	-2.21776189	1.65891230	-0.65714200
N	-3.14689757	1.85335525	1.39294692
H	-4.06484803	2.02726072	1.77676979
C	-1.97842041	1.46480376	2.15683378
H	-1.17207732	2.13248067	1.85355908
C	-1.63131326	0.00015489	1.92676285
O	-0.45704635	-0.35993353	1.97619720

N	-2.58336239	-0.84155266	1.51829240
H	-3.54755084	-0.59393448	1.34751765
C	-2.20765685	-2.21013553	1.22517026
H	-1.64794357	-2.62648270	2.06196788
C	-1.29409454	-2.31670016	0.01187342
O	-0.42950901	-3.18069515	-0.11056330
N	-1.46135357	-1.41823151	-0.96095583
H	-2.22340242	-0.76131103	-0.87449675
C	-0.61296294	-1.30591021	-2.13000783
H	-0.19043372	-2.30666324	-2.22042135
C	0.64120749	-0.46071405	-1.95256675
O	1.73304092	-0.95250086	-2.22758622
N	0.50536322	0.71592243	-1.33808472
H	-0.41417840	0.95273905	-0.99413234
C	1.66997775	1.46704768	-0.91468753
H	2.26565550	1.70647141	-1.79544939
C	2.62743108	0.73335836	0.01339998
O	3.80976824	1.02119336	-0.16099638
N	2.22985639	-0.23730233	0.83983173
H	1.26645913	-0.45200656	1.05263672
C	3.17023476	-0.94723138	1.68389606
H	3.66811524	-0.26073337	2.36806540
C	4.25121532	-1.62078531	0.85082195
O	5.29139948	-1.91831255	1.43260859
N	3.95290023	-1.91727710	-0.41653079
H	3.07605834	-1.65045143	-0.83993899
C	4.90596704	-2.63596957	-1.23839822
H	5.12075990	-3.60475859	-0.78757339
C	6.14365880	-1.79950275	-1.52897134
O	7.21805255	-2.35242001	-1.75322736
N	6.04087586	-0.47461302	-1.39983141
H	5.12302545	-0.19585134	-1.08401711
C	7.02276848	0.58876115	-1.46035917
H	7.86193527	0.20634323	-2.04321145
C	7.62866367	0.85203426	-0.08902566
O	8.79305630	1.23117623	0.01454748
N	6.98109069	0.65927587	1.06267206
H	6.01433423	0.39832770	0.93603009
C	7.44286324	0.96941684	2.40056816
H	8.08946233	1.84394317	2.33263830
C	8.09151035	-0.24205201	3.05526310
O	8.79352160	-0.05004832	4.04562364
N	7.98422713	-1.41454558	2.42790166
H	7.40896256	-1.40924157	1.59716240
C	8.81175202	-2.57717369	2.68189925
H	9.11735358	-2.60367914	3.72799262
H	-11.11883744	-1.64969964	1.67066595
H	-10.24497747	-1.37811210	0.14918844
H	-6.71649034	0.91882707	2.64587746
H	-2.21536630	1.63037771	3.18704828
H	-4.51780543	3.30380568	-0.94196621
H	-1.22115220	-1.09346480	-2.98433374
H	-3.10340263	-2.78861261	1.13626710
H	1.28742746	2.36388360	-0.47396015
H	2.66066461	-1.66745366	2.28930346
H	4.53464932	-2.91872711	-2.20124365
H	9.76286975	-2.58559624	2.19179302
H	8.31009572	-3.49586336	2.45997526
H	6.60543037	1.50067637	-1.83337330
H	6.65329419	1.23283054	3.07294747

pDIQ-c1

C	-9.71619341	-4.11156759	0.59538196
H	-9.07460870	-4.54376859	-0.17355924
C	-8.77392093	-3.44749545	1.58854112
O	-9.04117317	-3.41292316	2.78791896
N	-7.66739236	-2.89321076	1.08890893
H	-7.66313999	-2.96466732	0.08137380
C	-6.68516207	-2.07710102	1.77432475
H	-7.13549815	-1.55979599	2.62141513
C	-6.31637125	-0.86587882	0.92879330
O	-6.45276049	-0.98773489	-0.28639981
N	-5.94600316	0.28605317	1.49281229
H	-5.72619934	0.34427876	2.47647748
C	-5.27522463	1.34593273	0.76691299
H	-5.89715878	1.52671637	-0.11009464
C	-3.90192280	0.96481620	0.23349136

O	-2.81833888	1.33339312	0.68058210
N	-3.92338779	0.34166050	-0.94805444
H	-4.84222351	0.02336048	-1.21845260
C	-2.79222401	-0.23321585	-1.64770578
H	-2.32044462	-0.94959172	-0.97454015
C	-1.80679963	0.83646393	-2.09523490
O	-0.59144317	0.65156887	-2.11406888
N	-2.33237700	2.04312276	-2.32083661
H	-3.32146514	2.19033239	-2.17730914
C	-1.57159373	3.25416620	-2.55175847
H	-1.21186787	3.18804965	-3.57800458
C	-0.43185078	3.53080280	-1.58069332
O	0.57862664	4.12892953	-1.94219853
N	-0.69631022	3.12467834	-0.33709314
H	-1.52444094	2.58442435	-0.12702583
C	0.17038114	3.29323726	0.81110208
H	1.02265142	3.91296008	0.53141444
C	0.86847322	1.99073569	1.17558452
O	2.07903752	2.04240891	1.38227548
N	0.11031344	0.89321056	1.13611827
H	-0.82954084	0.94292121	0.76800425
C	0.67568197	-0.37165313	1.55956555
H	1.15661618	-0.23398165	2.52822133
C	1.81442738	-0.83556425	0.66353894
O	2.97069482	-0.89653271	1.07713497
N	1.51842414	-0.74528796	-0.63586959
H	0.55877822	-0.52501078	-0.85950055
C	2.50634389	-1.16828736	-1.60704176
H	2.75010222	-2.21611487	-1.43547383
C	3.80015225	-0.38032856	-1.75143780
O	4.69876027	-0.97451734	-2.34380048
N	3.92404729	0.86106640	-1.27650535
H	3.07850725	1.11919270	-0.78901792
C	5.08672298	1.72447696	-1.22824818
H	5.64786703	1.69933467	-2.16237578
C	6.02316419	1.31373793	-0.10064159
O	7.23885938	1.29950153	-0.28206868
N	5.54517791	0.84760708	1.05543988
H	4.54424556	0.86724236	1.18669945
C	6.36533538	0.19710740	2.05691189
H	7.21815326	0.87539325	2.05488965
C	6.87290733	-1.15291638	1.57105919
O	7.95707562	-1.53717545	2.00401796
N	6.07102891	-1.83128863	0.74699773
H	5.12303661	-1.52403485	0.57846909
C	6.27789636	-3.24942633	0.52974760
H	6.81640464	-3.69390213	1.36636237
C	7.25864003	-3.35263984	-0.62918907
O	8.16568973	-4.18176041	-0.61066644
N	7.21913515	-2.51363513	-1.66690229
H	6.46733991	-1.84425996	-1.75181363
C	8.13218837	-2.43999507	-2.78991087
H	8.15562631	-3.40853692	-3.29028326
H	-10.32156066	-4.89427487	1.00256719
H	-10.33164292	-3.38309215	0.11015476
H	-5.80962460	-2.59156682	2.11145836
H	-5.28979317	2.23809706	1.35744712
H	-3.20603912	-0.81759835	-2.44278700
H	-2.29324340	4.04018276	-2.47238764
H	-0.43339577	3.70606359	1.59208086
H	-0.10422414	-1.10298884	1.60192742
H	4.86771729	2.75016475	-1.01634946
H	2.09236209	-1.11215661	-2.59211455
H	7.84662042	-1.70929152	-3.51752565
H	9.14300275	-2.22447205	-2.51295579
H	5.32110759	-3.69558213	0.35539089
H	5.86620637	0.18277285	3.00325472

pDIQ-c2

C	10.61340846	-2.79513234	-2.93418668
H	9.86675561	-3.26426392	-3.57580043
C	9.99052620	-1.50865584	-2.41131268
O	10.65471630	-0.56238991	-1.99535297
N	8.68463529	-1.29431046	-2.58459623
H	8.10301456	-2.02488044	-2.96820537
C	7.89357023	-0.11595321	-2.29177614
H	8.56382587	0.72997152	-2.13966462

C	7.13994446	-0.29356370	-0.98174408
O	6.42835521	-1.28045452	-0.80861552
N	7.10571839	0.71009532	-0.10219624
H	7.72180834	1.49638998	-0.25309685
C	6.29684985	0.83873848	1.09314512
H	5.74242851	-0.08759303	1.24127713
C	5.13203908	1.81374841	0.99577546
O	4.15841034	1.60698873	1.71585999
N	5.27122299	2.74971581	0.05391606
H	6.21559045	2.84750295	-0.29028579
C	4.34300736	3.84657242	-0.13394191
H	4.38069225	4.47223559	0.75860480
C	2.88209590	3.48578445	-0.36287002
O	2.09147159	4.42574818	-0.32395219
N	2.48821652	2.23246070	-0.60072686
H	3.18475261	1.53660426	-0.37427504
C	1.25251671	1.72952221	-1.16672169
H	0.81976376	2.60883152	-1.64384764
C	0.30378397	1.12396345	-0.14220077
O	-0.83214570	0.90031438	-0.55328482
N	0.60736777	1.09604576	1.15777682
H	1.52495669	1.39983069	1.44939718
C	-0.19226726	0.40868618	2.15151619
H	-0.25252386	-0.61691483	1.78850258
C	-1.63861042	0.85060116	2.32475576
O	-2.50111337	-0.02379001	2.26753700
N	-1.83837727	2.17023974	2.35677879
H	-1.08756889	2.83674054	2.46718527
C	-3.16946693	2.74294859	2.37635444
H	-3.54716243	2.50040367	3.37009397
C	-4.10864574	2.23919665	1.28950004
O	-5.30627248	2.04483020	1.48796177
N	-3.52723302	1.88574875	0.14149478
H	-2.52381732	1.95378682	0.05460462
C	-4.32585737	1.32835092	-0.93192225
H	-5.26334141	1.88466251	-0.92378222
C	-4.72035149	-0.13994928	-0.86467457
O	-5.61059977	-0.53887504	-1.61202154
N	-4.21803023	-0.84715477	0.15002144
H	-3.59144543	-0.41272201	0.81319096
C	-4.45738653	-2.27007153	0.28040074
H	-4.33564030	-2.70408466	-0.71153405
C	-5.93110497	-2.48908654	0.59210046
O	-6.51851976	-3.45358823	0.10690197
N	-6.54822375	-1.63885670	1.41585761
H	-6.02448983	-0.94250389	1.92691991
C	-7.93737557	-1.69926637	1.82201807
H	-8.11073219	-2.67820921	2.26859232
C	-8.91560761	-1.50571179	0.67233421
O	-10.07709896	-1.90513655	0.71842031
N	-8.36114299	-0.82961290	-0.33564126
H	-7.39609419	-0.57546706	-0.17943700
C	-8.89454101	-0.28777173	-1.56899199
H	-9.97656629	-0.27280784	-1.44023048
C	-8.67300217	-1.07435734	-2.85342142
O	-9.33190939	-0.85980253	-3.86855185
N	-7.77944003	-2.06129693	-2.76325371
H	-7.27105098	-2.18065126	-1.89903773
C	-7.21644147	-2.81396141	-3.86594817
H	-7.57915226	-2.40566963	-4.80930329
H	11.51834576	-2.55835437	-3.45372943
H	10.76434498	-3.41298920	-2.07373850
H	7.24827921	0.09759790	-3.11815101
H	6.88356759	1.12731324	1.94013207
H	1.43826505	1.08562142	-2.00086157
H	4.75385032	4.46693074	-0.90287858
H	0.25004485	0.42136029	3.12573349
H	-3.83340377	1.45149527	-1.87384843
H	-6.15237910	-2.71204527	-3.81815315
H	-7.49696540	-3.84431640	-3.93358631
H	-8.11249842	-1.00019779	2.61292585
H	-3.11953363	3.80736032	2.27922397
H	-3.78184951	-2.72275211	0.97583588
H	-8.63559052	0.75042109	-1.56956145

1YCR

C	11.20312856	2.55570272	2.01448331
C	11.17815902	1.61374770	0.81474808
O	11.62293556	1.97190299	-0.27175264
N	10.72113329	0.38997622	1.02008946
C	10.67964849	-0.54958692	-0.07990346
C	9.31730214	-0.51521961	-0.77621492
O	8.33970173	0.01005448	-0.22193358
N	9.26257632	-1.07380652	-1.98784482
C	8.03965913	-1.08659026	-2.76873939
C	6.88973591	-1.66485989	-1.98382778
O	5.85504410	-1.02480149	-1.84507160
N	7.08759343	-2.87351638	-1.46433270
C	6.06890939	-3.57307799	-0.69822392
C	5.51030100	-2.78565750	0.47532072
O	4.32170421	-2.86637838	0.75152350
N	6.35821938	-2.02317008	1.15625365
C	5.91541851	-1.19949128	2.26340584
C	5.03094745	-0.09934903	1.70659414
O	3.94559426	0.16223347	2.22206701
N	5.50604858	0.54367937	0.64412971
C	4.79502352	1.64592602	0.02869165
C	3.44903421	1.24690776	-0.55966413
O	2.44464883	1.94628490	-0.36642117
N	3.43179099	0.11545863	-1.25622411
C	2.24101750	-0.38629407	-1.94620021
C	1.11359739	-0.85005307	-1.02609270
O	-0.05704281	-0.83042654	-1.39020470
N	1.45507068	-1.27373972	0.17109186
C	0.42837177	-1.72143522	1.08440199
C	-0.34108171	-0.56266677	1.71517403
O	-1.39130556	-0.77151851	2.31692316
N	0.13790837	0.65924141	1.52018135
C	-0.53726066	1.83191848	2.06398790
C	-1.58357967	2.40808687	1.12138520
O	-1.95696198	3.56978710	1.23943793
N	-2.02454663	1.62050392	0.15585814
C	-3.01834605	2.08294339	-0.80572948
C	-4.39623121	1.67748143	-0.31176955
O	-4.53731236	0.60231388	0.28834383
N	-5.43658042	2.49646120	-0.62035012
C	-6.82963524	2.25547339	-0.22479312
C	-7.23875937	0.86675188	-0.69117638
O	-7.24576970	0.58176833	-1.89103989
C	-7.58029428	3.34839727	-0.96950701
C	-6.57831324	4.44729622	-1.03234358
C	-5.35659861	3.71066354	-1.44976069
N	-7.55505554	0.00909586	0.27630394
C	-7.91609026	-1.38751325	0.03381844
C	-9.35341494	-1.71646507	-0.34149849
O	-9.71073484	-2.88445233	-0.49253316
N	-10.16387710	-0.69121464	-0.52136467
C	-11.55625346	-0.86106771	-0.90787255
C	-12.47923580	-0.95997824	0.32043591
C	-12.60711349	-2.39371598	0.86420123
O	-12.49118139	-3.38689452	0.12507853
N	-12.87869826	-2.49909080	2.16057968
H	10.90664119	2.08593598	2.96481616
H	11.47655196	-0.27627245	-0.78726432
H	10.21325791	0.16522846	1.86600371
H	7.73797550	-0.06897503	-3.06028096
H	10.13299781	-1.32977754	-2.44144492
H	5.20063705	-3.82161981	-1.32744022
H	7.97285908	-3.33550323	-1.63673550
H	5.31098813	-1.78139998	2.97087229
H	7.29483353	-1.88046669	0.79782242
H	4.57713622	2.43854695	0.75971681
H	6.40916555	0.29315914	0.25246927
H	1.80039827	0.39270499	-2.58627165
H	4.29771675	-0.40110957	-1.37642334
H	-0.31430944	-2.34175862	0.56494930
H	2.42857433	-1.30848250	0.46497882
H	-1.04072476	1.55474651	3.00263361
H	0.98676461	0.82419570	0.98433304
H	-2.89416213	3.16470411	-0.91610783
H	-1.76519817	0.64140831	0.11462974
H	-8.52411826	3.61644208	-0.46769729

H	-7.82004422	2.97819859	-1.97909843
H	-6.85281458	5.25297483	-1.73264634
H	-6.42546283	4.90723213	-0.04050211
H	-5.37945150	3.45099990	-2.52444952
H	-4.43308590	4.27081534	-1.25176808
H	-6.93061289	2.35678375	0.87159807
H	-7.29652864	-1.77748801	-0.79058455
H	-7.26034059	0.24830669	1.21575613
H	-13.50306307	-0.65700060	0.03253465
H	-12.15217018	-0.25554379	1.10468267
H	-12.91443148	-1.70280797	2.78271516
H	-12.97649387	-3.42943491	2.55152202
H	-11.64507549	-1.79263814	-1.48552981
H	-9.81134054	0.24482005	-0.36303619
H	-11.84934294	-0.00795849	-1.53804075
H	-7.66743379	-1.97933124	0.92586749
H	-2.84100662	1.59451979	-1.77833819
H	0.19194238	2.62848029	2.26241164
H	0.88894357	-2.31359269	1.88852861
H	2.54763392	-1.22904664	-2.58429320
H	6.50009106	-4.51629475	-0.32989644
H	6.79510208	-0.78040388	2.77341743
H	5.43508152	2.05807193	-0.76636356
H	8.21223574	-1.67593000	-3.68236667
H	10.86563302	-1.57515478	0.28428968
H	12.22366102	2.95451841	2.10503113
H	10.53647170	3.40741227	1.80907608

3JZQ

C	7.51162231	4.53032062	0.42520171
C	7.86371796	3.15392589	-0.13537968
O	8.27821933	3.03155426	-1.28698467
N	7.66225320	2.11831427	0.66086573
C	7.94906975	0.77388977	0.21137232
C	6.75866987	0.19306292	-0.56492310
O	5.64589732	0.74083354	-0.54703781
N	7.00814757	-0.92694418	-1.23811419
C	5.94512584	-1.59939357	-1.96939728
C	4.80886554	-1.99021884	-1.00449808
O	3.60526190	-1.81820218	-1.29962987
N	5.17003244	-2.54537262	0.13909145
C	4.15362644	-2.94797595	1.09052862
C	3.25606731	-1.76056612	1.54100375
O	2.03210066	-1.88200205	1.64118445
N	3.86632177	-0.61139482	1.77533701
C	3.12924397	0.56905913	2.21521208
C	2.06288386	0.95056018	1.18318573
O	0.89950335	1.16786314	1.52833848
N	2.47947797	1.05587151	-0.07567174
C	1.56203547	1.38877151	-1.16245653
C	0.48209738	0.33128906	-1.35426354
O	-0.72218460	0.64931309	-1.45076151
N	0.89464272	-0.92934769	-1.41382500
C	-0.06704912	-2.00618411	-1.65233589
C	-1.09386500	-2.21783668	-0.55899575
O	-2.30068418	-2.42682660	-0.84646732
N	-0.62845356	-2.17898325	0.68991972
C	-1.52986275	-2.43322491	1.80473853
C	-2.63704790	-1.38820448	1.89081797
O	-3.64307798	-1.62051678	2.53382473
N	-2.45608040	-0.24730841	1.24902660
C	-3.48685950	0.76422785	1.35233758
C	-4.38152730	0.91615890	0.12977150
O	-5.16804535	1.86164443	0.03004343
N	-4.27374258	-0.01560988	-0.80902541
C	-5.16351114	0.03819177	-1.98226989
C	-6.57817164	-0.15198833	-1.42337672
O	-6.76600972	-0.96516017	-0.55000244
N	-7.56355402	0.55843006	-1.94875424
C	-8.95738139	0.42626811	-1.45875139
C	-9.20673765	1.02068817	-0.07222422
O	-10.14097748	0.60973871	0.64583541
N	-8.36491433	1.99120274	0.28250283
C	-8.45040884	2.73303395	1.53972694
H	8.07906167	4.74887634	1.34517682
H	8.83520860	0.80163467	-0.44231613
H	7.21233077	2.24648889	1.55819557

H	5.49806070	-0.94734389	-2.73356612
H	7.96908594	-1.22112746	-1.37547109
H	3.46836402	-3.68607188	0.64997371
H	6.15150602	-2.69454481	0.34177989
H	2.59940375	0.37262650	3.15673234
H	4.83920488	-0.50331905	1.51559792
H	1.03456045	2.33015870	-0.95368915
H	3.44991084	0.85852892	-0.30848378
H	-0.64222018	-1.81005284	-2.56893206
H	1.87949410	-1.16509634	-1.31957439
H	-2.03095424	-3.40734716	1.70672347
H	0.35678485	-2.00175677	0.88051990
H	-4.14099237	0.50731987	2.19685317
H	-1.60449028	-0.02641817	0.74157883
H	-5.03648218	0.98972835	-2.52405129
H	-3.64193349	-0.81019963	-0.70673826
H	-9.62829691	0.91563798	-2.18150290
H	-7.35500818	1.29094222	-2.61671058
H	-9.24118005	2.28155439	2.15409133
H	-7.48894178	2.08836418	-0.22732736
H	-8.69258041	3.79470431	1.35809990
H	-7.48706674	2.67562246	2.06999858
H	-9.22279521	-0.63750509	-1.39530543
H	-4.91757539	-0.80506165	-2.64223026
H	-3.04348414	1.75396445	1.54434471
H	-0.94926765	-2.41752914	2.73843081
H	0.50627696	-2.93708022	-1.78924308
H	3.84545367	1.39241659	2.35190473
H	2.15233006	1.49718101	-2.08447979
H	6.34957940	-2.50070771	-2.45276645
H	4.64094889	-3.39309631	1.97053808
H	6.43725375	4.58363113	0.66962091
H	7.74624501	5.27992641	-0.33902050
H	8.16964242	0.12390227	1.07488748

4N5T

C	10.49575649	-5.19596423	-0.44664848
O	11.08415206	-5.10304513	-1.50918211
C	10.94805851	-6.12862752	0.66251846
N	9.37389389	-4.45107981	-0.15976565
C	8.93786147	-3.39749895	-1.03480565
C	8.94688643	-2.04439001	-0.33299498
O	8.87428617	-1.92669572	0.88189818
N	8.98437668	-0.95004282	-1.14645267
C	8.85454981	0.35706378	-0.55490559
C	7.53998108	0.47850627	0.22483914
O	6.49036033	0.02612402	-0.22168933
N	7.58804852	1.18884378	1.38412595
C	6.42526180	1.22067916	2.25874256
C	5.16082474	1.76643370	1.58792036
O	4.06760159	1.28829982	1.86218758
O	2.22066877	2.27280716	-0.91090300
C	3.44083121	2.24507092	-0.81268098
C	4.21491753	3.35305977	-0.05794406
C	3.29110735	4.23916545	0.79265630
N	5.32859317	2.77477203	0.70117360
C	2.42912070	5.21055543	-0.02136065
C	1.57702707	6.12364272	0.86475390
C	0.74295119	7.15627878	0.09141539
C	-0.33839150	6.58978904	-0.84317782
C	-1.39241987	5.73032178	-0.13818983
C	-2.60930600	5.38233207	-1.00316413
N	4.19083718	1.28534288	-1.40551658
C	3.54047462	0.18308720	-2.08981202
C	2.69668145	-0.70609254	-1.16558085
O	1.58959739	-1.10020837	-1.50704922
N	3.25315683	-1.02078175	0.02888690
C	2.48152766	-1.75135622	1.01761847
C	1.19304725	-1.02167256	1.43661069
O	0.12772207	-1.61856473	1.52875617
N	1.32876939	0.30237104	1.68063667
C	0.16880012	1.10800945	2.00102471
C	-0.88480040	1.10589412	0.88404269
O	-2.07519253	0.97913468	1.14482109
N	-0.41057499	1.24677144	-0.37214735
C	-1.29870203	1.16410840	-1.51216077
C	-2.03597310	-0.17936632	-1.61952775

O	-3.20561407	-0.22017875	-1.98760251
N	-1.32591015	-1.27920255	-1.29483876
C	-1.94895862	-2.58749925	-1.31939648
C	-3.07871993	-2.74227640	-0.28696048
O	-4.16737815	-3.20231303	-0.60259431
N	-2.78514690	-2.34040002	0.97573318
C	-3.81852586	-2.39062517	1.99313209
C	-5.03721388	-1.50610021	1.66287299
O	-6.18079461	-1.88629541	1.86959764
C	-6.64736791	0.00341820	-0.42342226
N	-4.72926214	-0.30392204	1.12697544
O	-7.86416586	0.16261065	-0.39515473
C	-5.73464146	0.63412968	0.64655096
C	-4.96409436	1.84835765	0.11341138
C	-4.82679877	4.05479380	-1.09573308
C	-3.61371194	4.49444042	-0.26481238
C	-5.75573240	3.05943570	-0.37338194
N	-6.01674776	-0.70702690	-1.38737985
C	-6.75688771	-1.35219811	-2.44922239
C	-7.68305849	-2.50037211	-2.00783674
O	-8.64111988	-2.81238798	-2.69748538
N	-7.34233840	-3.10620746	-0.84117444
C	-8.18156817	-4.07969941	-0.18620746
C	-8.87045466	-3.58547824	1.09826156
O	-9.22306345	-4.39187443	1.94911921
N	-9.08664510	-2.24923592	1.16823259
C	-9.51584103	-1.61202585	2.39061994
H	9.59773841	-3.40328469	-1.91658418
H	9.01741581	-4.42846653	0.78986012
H	9.71267814	0.57788014	0.10273881
H	8.99652389	-1.05444634	-2.15331545
H	6.16426782	0.21453263	2.62322472
H	8.49842236	1.33629961	1.80726160
H	2.85448215	0.55621335	-2.86192410
H	5.16098217	1.15734399	-1.12861447
H	2.16374929	-2.72990791	0.63031768
H	4.15444358	-0.62856488	0.28239612
H	-0.34674642	0.74062349	2.90104426
H	2.25045007	0.73384971	1.64059432
H	-2.07780152	1.94023160	-1.47767478
H	0.58187658	1.41144606	-0.53136380
H	-2.40634065	-2.79685297	-2.29787798
H	-0.33046483	-1.20439058	-1.09436019
H	-3.75107872	-0.03658033	1.03001840
H	-7.41506884	-0.63936500	-2.96945877
H	-5.00001472	-0.76417478	-1.40091025
H	-8.96101006	-4.38734419	-0.90102221
H	-6.49034250	-2.82139944	-0.36663228
H	-10.05199665	-2.34883310	3.00480913
H	-8.64233137	-1.64544869	0.48103363
H	10.12530521	-6.44669755	1.32072004
H	11.70672867	-5.61397137	1.27541720
H	11.42262562	-7.00846815	0.20803191
H	3.93402615	4.81081793	1.48394829
H	2.65426105	3.59073959	1.41398318
H	3.08539318	5.83386621	-0.65852955
H	1.78579019	4.63472400	-0.70222407
H	2.23841259	6.65999286	1.56980354
H	0.91509644	5.50158931	1.49339456
H	0.25543426	7.82539632	0.82322906
H	1.42684870	7.79669962	-0.49543998
H	-0.84085917	7.43923568	-1.34012534
H	0.12553127	6.00491191	-1.65841351
H	-1.73783293	6.25481153	0.77304119
H	-0.93117734	4.78844595	0.21323745
H	-3.09998474	6.31287730	-1.34500114
H	-4.21386173	-3.40998966	2.11586289
H	-4.28136906	2.15642530	0.91791835
H	-4.32274068	1.49007915	-0.70799853
H	-5.40085100	4.94494146	-1.40965102
H	-4.46520472	3.58084974	-2.02839572
H	-3.07114981	3.60374183	0.09349804
H	-6.25448206	3.55401990	0.48006720
H	-6.56156690	2.74571795	-1.05790170
H	-10.18270374	-0.76342093	2.16860256
H	-8.64663359	-1.23918647	2.96286313
H	-7.60860870	-4.97469021	0.10068781

H	-1.16896580	-3.33697360	-1.11853854
H	-6.41977841	0.92003313	1.46159878
H	-3.38165425	-2.06288376	2.94917042
H	-6.03502765	-1.75147722	-3.17762207
H	-0.69919620	1.31579083	-2.42257685
H	0.50223469	2.14127831	2.18326536
H	4.31733751	-0.43011948	-2.57075906
H	3.11364805	-1.90968839	1.90440471
H	4.67924883	3.97876976	-0.83948213
H	6.65799062	1.84987405	3.13173020
H	8.83946355	1.11330560	-1.35497484
H	7.90284887	-3.56420608	-1.39356623
H	6.26571009	3.13127926	0.55435731
H	-1.86452704	-1.96215238	1.19349406
H	-2.27021172	4.86513694	-1.92133317
H	-3.96420325	5.01451460	0.64707944
